# Comparative Analysis of Fruit Ripening-Related miRNAs and Their Targets in Blueberry Using Small RNA and Degradome Sequencing

**DOI:** 10.3390/ijms18122767

**Published:** 2017-12-19

**Authors:** Yanming Hou, Lulu Zhai, Xuyan Li, Yu Xue, Jingjing Wang, Pengjie Yang, Chunmei Cao, Hongxue Li, Yuhai Cui, Shaomin Bian

**Affiliations:** 1 College of Plant Science, Jilin University, Changchun 130062, China; houym15@mails.jlu.edu.cn (Y.H.); zhailulu@jlu.edu.cn (L.Z.); xuyanli@jlu.edu.cn (X.L.); wangjj8214@mails.jlu.edu.cn (J.W.); yangpj8214@mails.jlu.edu.cn (P.Y.); caocm8214@mails.jlu.edu.cn (C.C.); lihx8214@mails.jlu.edu.cn (H.L.); 2 College of Life Sciences, Jilin University, Changchun 130012, China; leucinepot@163.com; 3 Agriculture and Agri-Food Canada, London Research and Development Centre, London, ON N5V 4T3, Canada; yuhai.cui@agr.gc.ca

**Keywords:** blueberry, fruit ripening, miRNA, sRNA sequencing, targets, degradome sequencing

## Abstract

MicroRNAs (miRNAs) play vital roles in the regulation of fruit development and ripening. Blueberry is an important small berry fruit crop with economical and nutritional value. However, nothing is known about the miRNAs and their targets involved in blueberry fruit ripening. In this study, using high-throughput sequencing of small RNAs, 84 known miRNAs belonging to 28 families and 16 novel miRNAs were identified in white fruit (WF) and blue fruit (BF) libraries, which represent fruit ripening onset and in progress, respectively. Among them, 41 miRNAs were shown to be differentially expressed during fruit maturation, and 16 miRNAs representing 16 families were further chosen to validate the sRNA sequencing data by stem-loop qRT-PCR. Meanwhile, 178 targets were identified for 41 known and 7 novel miRNAs in WF and BF libraries using degradome sequencing, and targets of miR160 were validated using RLM-RACE (RNA Ligase-Mediated (RLM)-Rapid Amplification of cDNA Ends) approach. Moreover, the expression patterns of 6 miRNAs and their targets were examined during fruit development and ripening. Finally, integrative analysis of miRNAs and their targets revealed a complex miRNA-mRNA regulatory network involving a wide variety of biological processes. The findings will facilitate future investigations of the miRNA-mediated mechanisms that regulate fruit development and ripening in blueberry.

## 1. Introduction

Blueberry is an economically and nutritionally important small fruit crop, and commercially harvested blueberry species include lowbush (*Vaccinium angustifolium*), highbush (*V. corymbosum*), and rabbiteye bluberry (*V. ashei* or *V. virgatum*). Blueberry fruit not only tastes sweet with variable acidity, but also is an especially rich source of polyphenolic anthocyanin pigments [1], which contribute to recognized health-promoting effects including improvement of night vision, prevention of macular degeneration, anti-cancer activity, and reduced risk of heart disease [2]. For further genetic improvement of the crop, it is crucial to elucidate the molecular mechanisms that control fruit development and biosynthesis of anthocyanins in blueberry.

The developmental process of blueberry fruit after fruit ripening onset can be conceptually divided into two sequential phases: expansion and maturation. In the expansion phase, young fruit is hard and dark green with varied sizes. Subsequently, enlarged fruit begins to soften and accumulate red and then blue pigments in the maturation phase, which is further divided into different stages by fruit colors (light green/white, pink and blue stage) [3]. In recent years, RNA sequencing has become a powerful technology to profile the transcriptome and has provided much valuable information for gene identification and their potential roles during blueberry fruit development and maturation [3,4,5,6]. However, additional regulators need to be discovered to better understand the developmental regulatory network that controls fruit development and ripening.

MicroRNAs (miRNAs) are a class of endogenous, 20–24 nucleotides (nt) in length, single-stranded, non-coding RNAs which act as important regulatory molecules found in diverse eukaryotes [7]. Generally, *MIR* genes can be transcribed into primary transcripts (pri-miRNA), which are subsequently processed into the stem-loop precursor (pre-miRNA) by a DCL (Dicer-like) protein. The pre-miRNA is further processed by DCL1 into a duplex of miRNA and miRNA* [8]. In plants, miRNA can bind to transcripts of target genes based on perfect or near-perfect pairing, leading either to cleavage-induced degradation of the mRNA or translational inhibition [9,10].

Plant miRNAs act as master regulators implicated in regulating various cellular and metabolic processes [7,11,12,13,14]. Recently, emerging evidence has demonstrated that miRNAs play an important role in the regulation of fruit development and maturation. For example, abnormal fruit morphologies can be generated by over-expression of *AtMIR156b* in tomato [15], while small tandem target mimic-mediated blockage of miR858 promoted anthocyanin accumulation in tomato [16]. miR156/157 and miR172 were shown to control the ripening process of tomato via targeting the known ripening regulators *CNR* and *AP2a*. Similarly, miR159 acts as a ripening regulator by targeting *FaGAMYB*, which plays crucial roles during the transition of strawberry receptacle from development to ripening [17]. Most recently, it was reported that miR73 was involved in regulation of strawberry fruit ripening via targeting *ABI5* transcript to affect ABA (Abscisic acid) signaling pathway [18]. With the application of the high-throughput sequencing technology in miRNA-related research, numerous miRNAs involved in fruit development and maturation have been identified in many fruit-producing species such as apple [19], grape [20,21], peach [22,23], pomegranate [24], date palm [25], sweet orange [26]. Meanwhile, degradome sequencing has been developed for genome-wide identification of miRNA target genes and has been successfully applied to reveal miRNA-mRNA target pairs during fruit development [19,26]. Thus, the combinatorial analysis of miRNAome and degradome has greatly enhanced our understanding of the roles of miRNA during fruit development.

The blueberry genome is large (600 Mb/haploid genome) [27] and genomic information is limited. We previously reported the computational identification of conserved miRNAs and their targets from expression sequence tags in blueberry [28]. Recently, the availability of the genome sequence of cranberry (closely related to the species of blueberry) and blueberry transcriptome data makes it possible to effectively identify miRNAs and their targets in blueberry at genome level. In this study, a combinatorial analysis of miRNAome and degradome sequencing was conducted to identify and characterize the miRNAs and their targets that act during blueberry fruit maturation, a key developmental phase for fruit quality and biosynthesis of anthocyanin. The libraries for miRNAome and degradome profiling were made from fruits at white and blue fruit stages, which represent fruit ripening onset and in progress, respectively. The findings provide new insights into understanding the regulatory network that controls fruit ripening in blueberry.

## 2. Results

### 2.1. Global Analysis of Sequences from Small RNA and Degradome Libraries

Fruit maturation is a very important phase in the developmental process of blueberry fruit, and many complex biochemical changes occur during the phase such as softening, sweetening, cell enlargement and pigmentation. To identify miRNAs involved in the ripening of blueberry fruits, two small RNA libraries were constructed for sequencing from samples of white (WF) and blue fruits (BF), which represent ripening onset and progression, respectively. Many biological changes involved in fruit ripening occur between these two stages such as fruit softening, coloration [6]. In total, approximately 13.89 and 31.40 million raw reads representing 3.08 and 5.99 million unique sequences were generated from WF and BF, respectively (Table S1). After removing the low quality reads, 3′ adapters, junk reads, and redundant repeats, 4.58 and 9.56 million clean reads were obtained from the two libraries, including 1.53 and 2.38 Rfam RNAs (rRNA, tRNA, snoRNA and snRNA, Table S1), which showed high quality sequencing data in this study. The small RNA sequences with length of 18–25 nucleotides accounted for 66.66% (WF) and 75.09% (BF) of all the clean reads, respectively. Among them, 24 nt small RNAs are the most abundant in both libraries, followed by 21 nt (Figure S1). Moreover, a statistical analysis of the nucleotide bias for all locations (1–25) showed that sRNAs starting with a uridine (U) at the 5′ end are commonly present in blueberry fruit, and the U is also most frequently observed at the 24th nucleotide (Figure S2).

To further demonstrate the biological functions of miRNAs identified in blueberry, degradome sequencing technology was adopted to perform transcriptome-wide analysis of the miRNA-cleaved mRNAs. A total of 10,686,450 and 13,702,581 raw reads were obtained from the two degradome libraries (WF and BF), respectively (Table S1). Approximately 4.49 and 4.96 million unique sequences were successfully mapped to the *V. corymbosum* reference sequences (transcriptome and expressed sequence tags), accounting for 99.31% of all unique reads (Table S1). Among them, more than 55% reads were mapped to cDNA sequences of blueberry. All mapped sequences were subsequently applied to detect candidate targets of miRNAs.

### 2.2. Identification of Fruit Ripening-Responsive miRNAs in Blueberry

**Figure 1:**
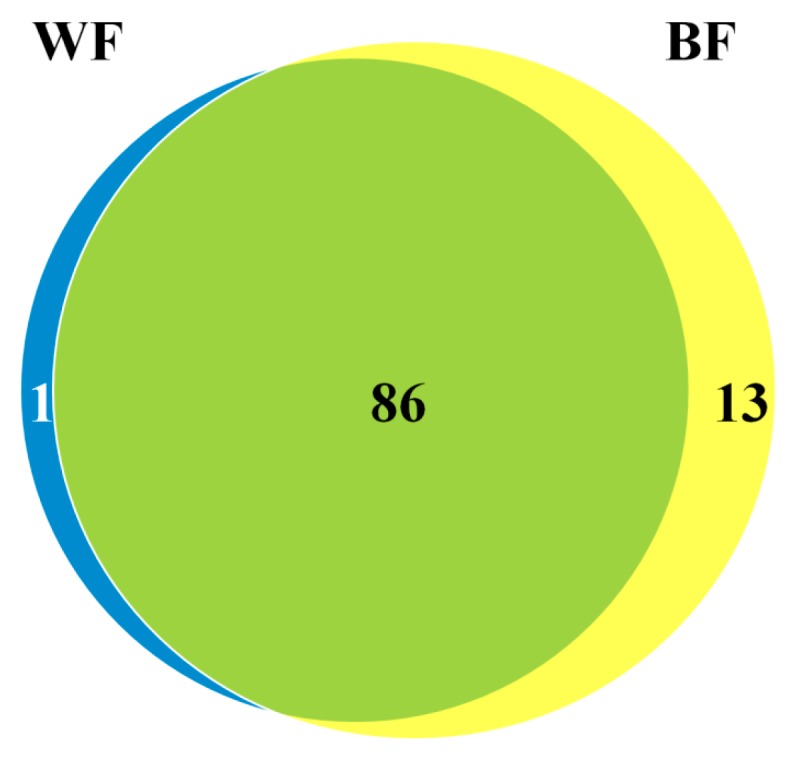
Venn diagram of blueberry miRNAs in the libraries of white (WF) and blue fruits (BF).

To identify the miRNAs responsive to fruit ripening, the valid sequences of sRNAs from the libraries of white (WF) and blue fruits (BF) were aligned with the known plant miRNAs in miRBase 21.0 or the *Vaccinium* genome. Following strict screening criteria for annotating miRNA sequence, a total of 84 known miRNAs belonging to 28 families and 16 novel miRNAs were identified in blueberry fruit (Table 1). Among them, 70 known miRNAs (83.3%) and 16 novel miRNAs (100%) were detected in both libraries, whereas 13 miRNAs were detected only in BF library, and one miRNA in WF (Figure 1), suggesting their specific roles in the regulation of fruit ripening.

The majority of the 84 known miRNAs are conserved across plant species (at least 12 species) based on the data from miRBase 21.0 such as miR156/157, miR159, miR160, miR165/166, miR172, miR319, miR396s, miR2111 and miR2118. Also, 7 less-conserved miRNA families were identified in blueberry fruit, including miR845, miR894, miR2603, miR6149, miR6478, miR7122 and miR7693, and the data from miRBase 21.0 indicated that they are present in only 1–5 plant species. Notably, the member numbers of each miRNA family varied in blueberry fruit. For example, miR156/157, miR166 and miR396 family contained 11, 10 and 13 members, respectively; while only one member was detected for 16 miRNAs families, including miR159, miR162, miR164, miR172, miR394, miR395, miR2111 and some less-conserved miRNA families (Table 1).

Sixteen novel miRNA sequences derived from 14 loci were identified in both libraries, namely vco-miR_n01 to vco-miR_n14. The characteristics of all the 16 novel miRNAs are summarized in Table 1. The length of these novel miRNAs varied from 19 to 24 nt, with the majority being 21 nt long (62.5%), which is consistent with the previous reports in plant [29]. The sequences of all the novel miRNAs were located on the stem-arm of secondary hairpin structure of their precursors. Among them, 5 of the novel miRNAs (miR_n01-02, miR_n08-5p, miR_n09, miR_n12-3p) began with a 5′ uridine, a characteristic feature of miRNAs. The precursors of the novel miRNAs ranged from 96 nt to 221 nt in length, which is acceptable since the lengths of miRNA precursors normally range from 55 to 930 nt in plants. They showed perfect or near-perfect stem-loop structures with no more than 2 mismatches in the miRNA/miRNA* duplex (Table 1 and Figure S3). It has been accepted that the minimal folding free energy index (MFEI) is an important criterion to differentiate the miRNA from other types of RNA [30]. In this study, the precursors of all the novel miRNA had perfect MFEIs (1.1–1.7) with an average of 1.4, which were higher than that of tRNAs (0.64), rRNAs (0.59) or mRNAs (0.62–0.66). The high MFEIs meet the requirements to maintain stability of secondary structure of miRNAs.

### 2.3. Phylogenetic Analysis of the Known and Novel miRNAs in Blueberry Fruit

**Figure 2:**
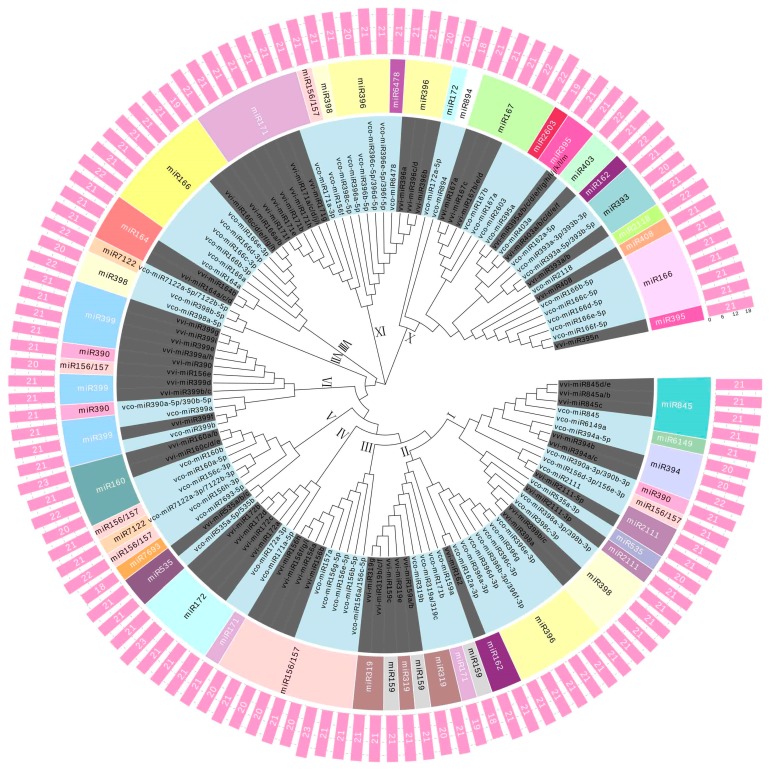
Phylogenetic analysis of known miRNA mature sequences of blueberry (*Vaccinium corymbosum*) and grape (*Vitis vinifera*) obtained from the sequencing data (this work) and miRbase 21.0, respectively. The miRNAs were clustered into 10 classes (I–X). The miRNAs in light blue background were from blueberry and the ones in dark grey were from grape in the inner ring. The middle ring shows the miRNA families in different colors. The outer ring shows the length of mature miRNAs.

To systematically exploit the evolutionary conservation of the known miRNAs in plant species, a phylogeny tree was constructed using the sequences of mature miRNAs in blueberry and grape, as they have a lot of commonalities in fruit property and developmental process. As shown in Figure 2, 84 blueberry miRNAs and 105 grape miRNAs were grouped into 10 classes, representing similarities and divergence. For example, all the members of the 9 miRNA families (miR395, miR166, miR408, miR2118, miR393, miR403, miR2603, miR167, miR894) from both blueberry and grape were clustered into class X, suggesting they were evolutionarily closer to each other. It was also observed that blueberry miRNAs were basically grouped together with their homologues in grape. For instance, all the vco-miR396-5ps were clustered together with vvi-miR396a/b/c/d in class IX, while all the vco-miRNA156/157-5ps and vvi-miR156a/b/f/g/h/i formed a discrete clade in the phylogenetic tree. These observations indicate that vco-miRNAs have close relationship with their corresponding homologs in grape. Meanwhile, the topological tree of miRNA precursors in blueberry and grape were constructed. In total, 55 and 93 precursors in blueberry and grape, respectively, were clustered into 5 classes, reflecting both conservation and divergence (Figure S4). As expected, miRNA precursors in blueberry showed close evolutionary relationship with their homologs in grape, further supporting the conservation of blueberry miRNAs.

To analyze the relationship of the newly identified vco-miRNAs with other miRNAs, their sequences were used as queries to search against all the miRNA sequences in miRBase 21 including animals, viruses and plants. As a result, one novel miRNA (vco-miR_n03) showed near-perfect sequence match with miR9202 (5 mismatches) from *Eptesicus fuscus*. Furthermore, the precursors of the 16 novel miRNAs were utilized to perform a BLASTX against the nucleotide sequence database at NCBI. The analysis revealed that homologs of all the precursors, except pre-vco-miR_n10, could not be found in any plant species other than those in the *Vaccinium* genus, suggesting that they might be *Vaccinium*-specific miRNAs in plants. An uncharacterized ncRNA in *Jatropha curcas* was found to be homologous to vco-miR_n10 with 82% identity.

### 2.4. Accumulation Patterns of Blueberry miRNAs during Fruit Ripening

It is generally accepted that the number of miRNA reads in a library can be considered as an indicator for estimating the relative abundance of miRNAs. In the present study, the normalized read counts of the known miRNAs varied from 0.42 to 1377.66 in BF library and 0 to 1055.95 in WF library. vco-miR166b-3p and vco-miR894 were the most abundant in BF and WF libraries, respectively (Table 1). Interestingly, different members in the same miRNA family also showed considerably variable abundance. In miR396 family, for example, the read number of miR396b-3p was 60.77 in BF library, whereas only 3.33 reads were detected for miR396c-3p. The variable abundance of miRNAs in each family might reflect their functional divergence during fruit maturation.

**Figure 3:**
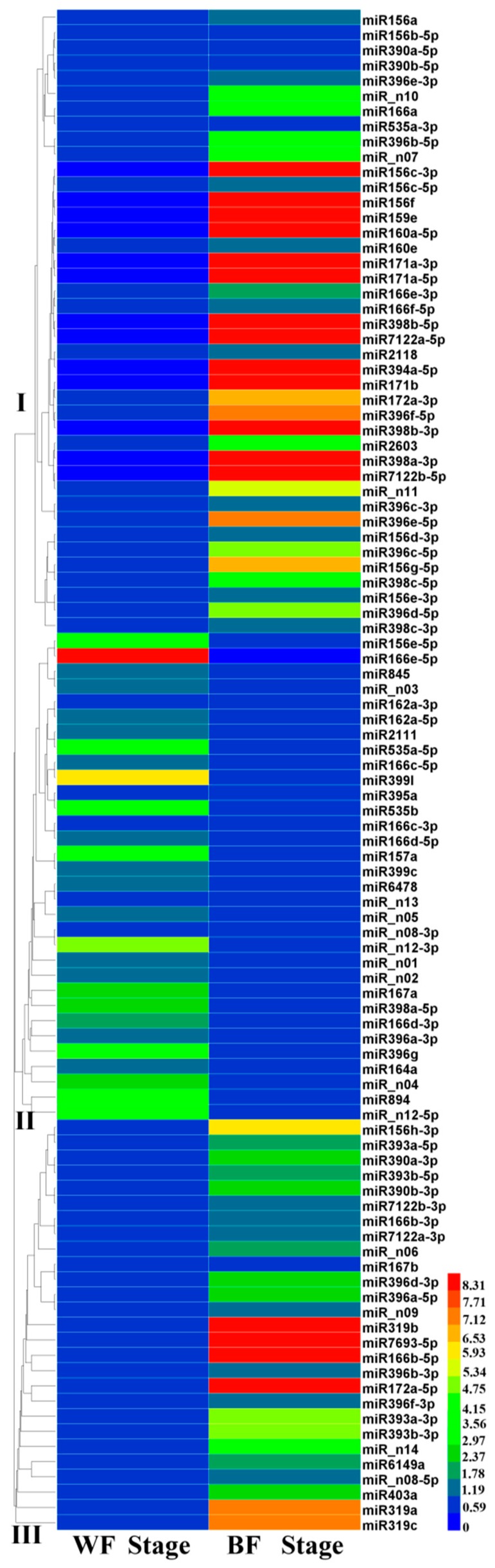
Expression analysis of known and novel miRNAs from blueberry obtained from the sequencing data. miRNAs were grouped into 3 classes (I–III) based on their accumulation patterns. WF and BF refer to white fruit and blue fruit, respectively.

To provide clues for their roles during fruit maturation, the abundance of the miRNAs in the two libraries were analyzed and compared using the normalized reads from the sRNA sequencing data. As shown in Figure 3, 100 miRNAs were categorized into three groups based on their accumulation patterns. Group II comprised 32 miRNAs with down-regulated accumulation as fruit ripens, whereas the rest of the miRNAs were up-regulated, which were separated into group I (38 known and 3 novel miRNAs) and III (23 known and 4 novel miRNAs) (Figure 3). It was also observed that different members within each miRNA family were conversely accumulated in the two libraries. For example, the accumulation of miR166a in BF library was increased by 3.0 folds, whereas miR166c-3p was decreased by 1.8 folds as compared to the one in WF library. These observations suggest that functional diversity of miRNAs might occur during fruit maturation, even for members within the same miRNA family.

To gain deeper insight into the roles of miRNAs during fruit maturation, we performed differential expression pattern analysis of known and novel miRNAs between the WF and BF libraries. The miRNAs were considered as up-regulated or down-regulated ones between the two libraries with the criteria of |log_2_(fold change)| ≥ 1 and *p*-value ≤ 0.05. A total of 35 known miRNAs belonging to 16 miRNA families and 6 novel miRNAs were differentially expressed during fruit maturation (Table 2). The majority of these miRNAs were up-regulated as fruit ripens. Among them, 11 miRNAs (vco-miR156c-3p/f, vco-miR159a, vco-miR160a-5p, vco-miR171a-3p/a-5p/b, vco-miR394a-5p, vco-miR398a-3p/b-3p/b-5p) were detected only in the BF library. Additionally, 3 miRNAs (vco-miR166b-5p, vco-miR319b and vco-miR7693-5p) was dramatically altered with 64.44, 16.82 and 13.86 fold changes, respectively. The up-regulation of miRNAs suggests that they might have positive correlation with fruit maturation via targeting repressor(s) of fruit ripening. Meanwhile, two miRNAs (vco-miR894 and vco-miR_n12-3p) were found to be down-regulated as fruit ripens, implying that they might be negatively responsive to fruit maturation.

**Figure 4:**
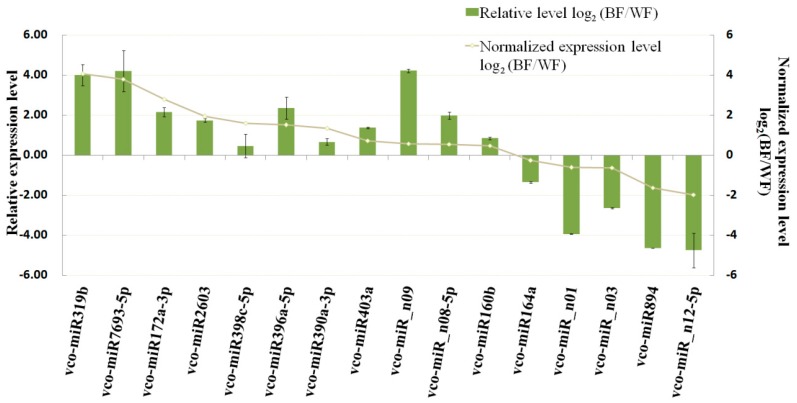
Expression comparison of selected miRNAs between WF and BF stages in blueberry by qRT-PCR and deep sequencing. Gray line represents transcript abundance changes calculated. Green bar indicates relative expression level determined by qRT-PCR analysis. The standard error of the mean is represented by an error bar. WF and BF refer to white fruit and blue fruit, respectively.

To validate the sRNA sequencing data, 16 miRNAs representing 16 families were randomly selected for analysis of their expression patterns using stem-loop qRT-PCR. The change tendency was calculated with log_2_(BF/WF), where BF/WF refers to the ratio of miRNA abundance in BF and WF libraries. As shown in Figure 4, the change tendency for each of the 16 miRNAs during ripening was quite consistent with the data from sRNA sequencing. The expression levels of the 11 up-regulated.

miRNAs (according to the sRNA sequencing data) were all increased, while the other 5 miRNAs (miR164a, miR894, miR_n01, miR_n03, miR_n12-5p) were shown to be down-regulated by both qRT-PCR and high-throughput sequencing. These observations indicated that the sRNA sequencing data presented here are highly reliable, and they could represent relative expression levels of the identified miRNAs in both blueberry libraries.

### 2.5. Identification of miRNA Targets during Fruit Ripening by Degradome Sequencing

**Figure 5:**
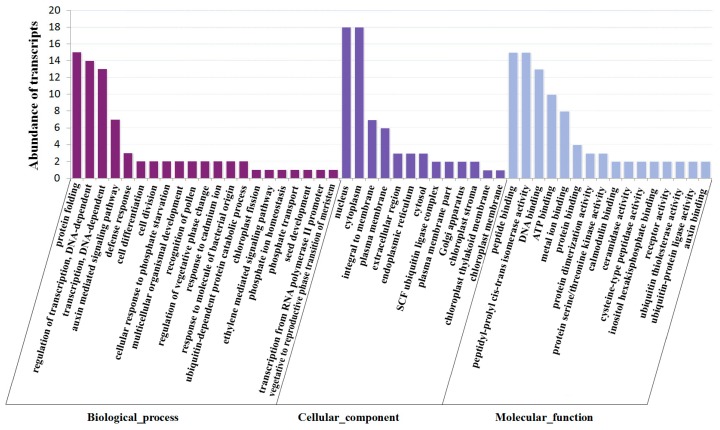
Gene ontology classification of target genes for differentially expressed known miRNAs between white fruit stage and mature fruit stage libraries in blueberry.

To understand the biological functions of miRNAs, it is essential to identify their targets. Therefore, a high-throughput degradome sequencing technology was employed to perform a transcriptome-wide analysis of the miRNA-guided cleavage of target mRNAs. As a result, a total of 178 sliced targets for 41 known and 7 novel miRNAs were identified in WF and BF libraries according to the CleaveLand pipeline (Tables S2 and S3). Furthermore, a confidence evaluation of the degradome data was conducted as described previously [29], and the sliced targets were grouped into 5 categories (0–4) accordingly. As exemplified in Figure 5, the abundance of miRNA-guided cleavage fragment is equal to the maximum on the transcript in category 0 and 1, while the cleavage fragment was not the most abundant tag, but it still formed a clear peak in category 2 and 3. Category 4 refers to only 1 raw read of miRNA-guided cleavage fragment detected in degradome sequencing. Category 0 is the most reliable for the detection of miRNA target genes, and 30 targets were included such as *SQUAMOSA PROMOTER-BINDING PROTEIN-LIKE* (*SPL*s), *AUXIN RESPONSE FACTOR* (*ARF*s), *CAP-Gly DOMAIN-CONTAINING LINKER PROTEIN*, *APETALA2* (*AP2*), *DISEASE RESISTANCE RPP13-LIKE PROTEIN*, *AUXIN SIGNALING F-BOX 2* (*AFB2*), *ARGONAUTE2* (*AGO2*), *TRANSPORT INHIBITOR RESPONSE 1* (*TIR 1*), and *PENTATRICOPEPTIDE REPEAT-CONTAINING PROTEIN* (PPR) (Tables S2 and S3). Meanwhile, 4 targets were detected in category 1, 90 targets in category 2, 10 targets in category 3 and 43 targets in category 4. Further observation indicated that the majority of the cleavage sites were distributed at the position between nucleotides 9 and 11 of the miRNA sequences from the 5′ to the 3′ end. Moreover, two cleavage targets of miR160b were selected for verification by RLM-5′ RACE (RNA Ligase-Mediated (RLM)-Rapid Amplification of cDNA Ends). As expected, the same cleavage site was detected for these two targets (Figure S5A,F), indicating a high reliability of the degradome data.

To better understand the functional roles of the miRNAs during fruit maturation, 92 cleaved transcripts caused by the differentially accumulated miRNAs in the two libraries were subjected to GO (Gene Ontology) functional analysis using Blast2GO. 57 targets were successfully assigned into 129 GO terms at the second level. By filtering out low abundance transcripts (copy numbers less than 2), the targets were shown to be enriched in 21 biological processes, 13 cellular components and 16 molecular functions (Figure 5). In biological processes, the predominant terms were related to protein folding (GO:0006457), regulation of transcription (GO:0006355), transcription (GO:0006351), and auxin-mediated signaling pathway (GO:0010928). Two GO terms, nucleus (GO:0005634) and cytoplasm (GO:0005737), were found to be predominant in cellular component category, while four terms were highly enriched in the molecular function including peptide binding, peptidyl-prolyl *cis*-*trans* isomerase activity, DNA binding, ATP binding (Figure 5). This analysis indicates that the targets identified by degradome sequencing might be involved in diverse biological processes during fruit maturation, especially DNA-dependent transcription, transcription regulation and protein folding.

**Figure 6:**
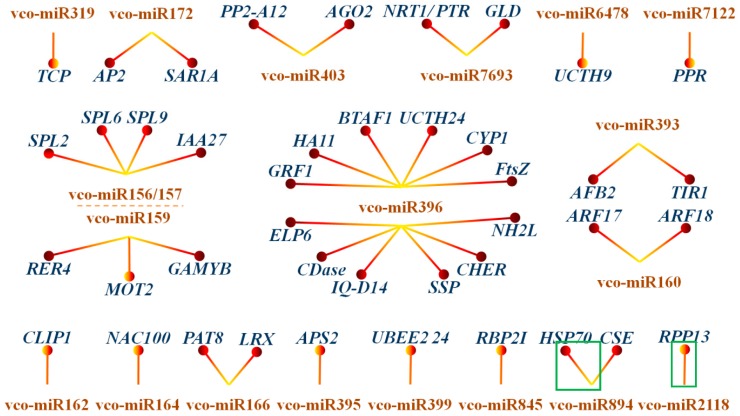
miRNA-mRNA regulatory network involved in blueberry fruit ripening. *TCP*, *TEOSINTE BRANCHED1/CYCLOIDEA/PROLIFERATING CELL NUCLEAR ANTIGEN FACTOR*; *AP2*, *APETALA 2*; *SAR1A*, *GTP-BINDING PROTEIN SAR1A-LIKE*; *PP2-A12*, *PHLOEM PROTEIN 2-A12*; *AGO2*, *ARGONAUTE 2*; *NRT1/PTR*, *NITRATE TRANSPORTER 1/PEPTIDE TRANSPORTER*; *GLD*, *GLUTAREDOXIN-C10*; *UCTH9*, *UBIQUITIN CARBOXYL-TERMINAL HYDROLASE 9*; *PPR*, *PENTATRICOPEPTIDE REPEAT-CONTAINING PROTEIN*; *SPL*, *SQUAMOSA PROMOTER-BINDING-LIKE PROTEIN*; *IAA27*, *INDOLE-3-ACETIC ACID 27*; *RER4*, *RETICULATA-RELATED 4*; *MOT2*, *MOLYBDATE TRANSPORTER 2*; *GAMYB*, *GIBBERELLIC ACID-REGULATED MYB*; *GRF1*, *GROWTH-REGULATING FACTOR 1*; *HA11*, *H(+)-ATPASE 11-LIKE*; *BTAF1*, *TATA-BINDING PROTEIN-ASSOCIATED FACTOR BTAF1*; *UCTH24*, *UBIQUITIN CARBOXYL-TERMINAL HYDROLASE 24*; *CYP*, *CYCLOPHILIN; FTSZ, FILAMENTING TEMPERATURE SENSITIVE Z*; *NH2L*, *NUDIX HYDROLASE 2-LIKE*; *CHER*, *CALCIUM HOMEOSTASIS ENDOPLASMIC RETICULUM PROTEIN-LIKE*; *SSP*, *SEED STORAGE PROTEIN*; *IQD14*, *IQ-DOMAIN14*; *CDase*, *NEUTRAL CERAMIDASE-LIKE*; *ELP6*, *ELONGATOR COMPLEX PROTEIN 6*; *AFB2*, *AUXIN SIGNALING F-BOX 2*; *TIR1*, *TRANSPORT INHIBITOR RESPONSE 1*; *ARF*, *AUXIN RESPONSE FACTOR*; *CLIP1*, *CAP-GLY DOMAIN-CONTAINING LINKER PROTEIN 1*; *NAC100*, *NAC DOMAIN-CONTAINING PROTEIN 100*; *PAT8*, *PROTEIN S-ACYLTRANSFERASE 8*; *APS2*, *ATP SULFURYLASE*; *UBC 24*, *UBIQUITIN-CONJUGATING ENZYME E2 24*; *RBP2I*, *RNA POLYMERASE II SECOND LARGEST SUBUNIT*; *HSP70*, *HEAT SHOCK 70 kDa PROTEIN 15-LIKE*; *RPP13*, *DISEASE RESISTANCE RPP13-LIKE PROTEIN 1*.

To gain insights into their functions, the miRNA targets detected by degradome sequencing were subjected to a BLASTX analysis to search for their homologs in plants. It was found that these targets encode transcription factors such as *SPL*, *MYB*, *TEOSINTE BRANCHED1/CYCLOIDEA/PROLIFERATING CELL NUCLEAR ANTIGEN FACTOR* (*TCP*), *AP2*, *GRF*, *NAC DOMAIN-CONTAINING PROTEIN* (*NAC*) family; enzymes such as *CYCLOPHILIN 1* (*CYP1*), *CERAMIDASE-LIKE* (*CDase*), *ATPase*, *ATP SULFURYLASE* (*APS*), *UBIQUITIN CARBOXYL-TERMINAL HYDROLASE 24* (*UCTH24*); structural proteins such as *SEED STORAGE PROTEIN* (*SSP*), *OLEOSIN 1*, *EXTENSIN*; chaperons (*HSP70*, *BAG*); transporters such as *MOLYBDATE TRANSPORTER 2* (*MOT2*), *NITRATE TRANSPORTER 1/PEPTIDE TRANSPORTER* (*NRT1/PTR*); and other regulators, which might be involved in different biological processes during blueberry fruit development and maturation. Furthermore, an integrative analysis of miRNAs and their targets was conducted to reveal the miRNA-mRNA regulatory network involved in blueberry fruit ripening. As shown in Figure 6, a number of conserved interactions between miRNAs and their targets across plant species were observed in blueberry such as vco-miR156-*SPL*s, vco-miR159-*GAMYB*, vco-miR172-*AP2*, vco-miR396-*GRF*s, vco-miR160-*ARF*s, vco-miR164-*NAC*, vco-miR403-*AGO2* and vco-miR319-*TIR1*. Besides, novel interactions were also identified in blueberry fruit. For example, miR159, targeting *GAMYB* in many plant species, could also target *RER4* and *MOT2* in blueberry, while *NRT1/PRT* and *GLD* (*GLUTAREDOXIN-C10*) were found to be targeted by the less-conserved miR7693. Intriguingly, 8 novel interactions were observed for the 7 novel miRNAs, which are involved in different biological processes. These novel interactions might be involved in the maintenance or regulation of blueberry fruit development in a species-specific manner. It was also observed that multiple genes were cleaved by a single miRNA. For instance, miR396 family (miR396a–g) can target 33 unique transcripts belonging to 12 different gene families. This observation suggested that the miRNAs might play diverse roles in the regulation of fruit development and maturation via targeting multiple genes in blueberry.

### 2.6. Dynamic Expression Patterns of Fruit-Related miRNAs and Their Targets during Fruit Development and Maturation

**Figure 7:**
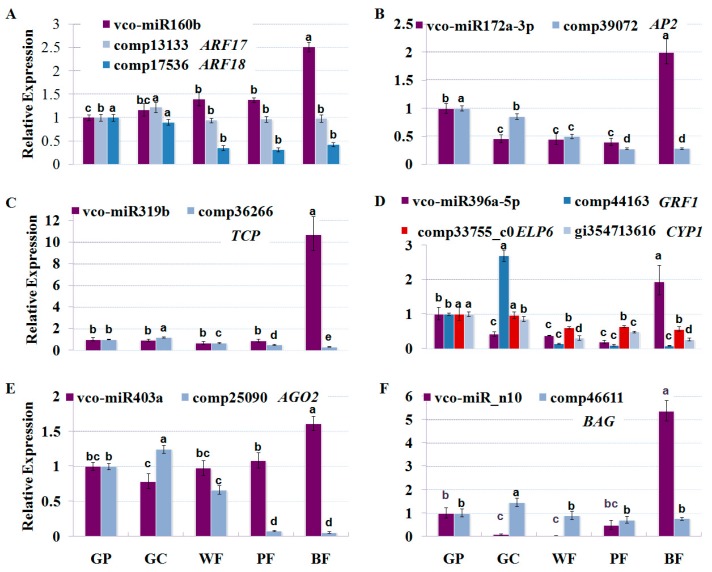
Expression patterns of the known and novel vco-miRNAs identified in this work and their target genes during fruit development and ripening. The accumulation patterns of vco-miR160b and 2 target genes (**A**), vco-miR172a-3p and its target gene (**B**); vco-miR319b and its target gene (**C**); vco-miR396a-5p and 3 target genes (**D**); vco-miR403a and its target gene (**E**); vco-miR_n10 and its target gene (**F**). The expression level in GP was set as 1. *U6* and *ACTIN* genes were used as the internal control for miRNA expression and target genes expression, respectively. Error bars indicate standard error of three biological and technical replicates. The different letters (a–d) indicate significant differences at *p* < 0.01 according to Duncan’s multiple range tests. GP, green pad; GC, green cup; WF, white fruit; PF, pink fruit; BF, blue fruit.

To investigate the dynamic expression patterns of the miRNAs at different developmental stages (green pad, green cup, white, pink and blue fruit) in blueberry, five known (vco-miR160b, vco-miR172a-3p, vco-miR319b, vco-miR396a-5p and vco-miR403a) and one novel (vco-miR_n10) vco-miRNAs were selected for stem-loop qRT-PCR analysis. As shown in Figure 7, these six miRNAs showed differential expression patterns during fruit development and maturation. miR160a was gradually increased as fruit develops and ripens, while 3 miRNAs (miR172a-3p, miR396a-5p and vco-miR_n10) were initially down-regulated, and remarkably elevated at blue fruit stage. miR319b and miR403a showed a stable accumulation at the initial four stages, and then a pronounced accumulation was clearly observed at blue fruit stage. Obviously, all the 6 miRNAs exhibited the highest accumulation at blue fruit stage, suggesting they might play crucial roles in the regulation of fruit maturation.

To confirm the regulatory roles of the vco-miRNAs, the expression patterns of 9 target genes corresponding to the 6 miRNAs were also investigated by qRT-PCR at the same developmental stages as above (Figure 7). For most target genes, their expressions were negatively correlated with those of their corresponding miRNAs. As shown in Figure 7, 8 target genes showed high expression at two fruit developmental stages (green pad and green cup stages), and decreased from onset of fruit ripening (white stage) until blue fruit stage. Especially, the expressions of miR396-targeted *GRF1* and miR160-targeted *ARF18* were decreased at white stage with 19.4 and 2.6 fold changes (Figure 7A,D), respectively, as compared to the ones at green cup stage, implying that they might play a role in the initiation of fruit maturation. It was also noteworthy that miR403-targeted *AGO2* was decreased at pink and blue fruit stages by 7.9 and 5.9 folds (Figure 7E), respectively, as compared to the ones at white stage, suggesting that it might be involved in the regulation of fruit maturation. The apparently negative correlation between the miRNAs and their target genes indicates that the miRNAs exert considerable influence on the expression of their target genes. Unexpectedly, *ARF17* showed a stable expression pattern during fruit development and maturation except at green cup stage with a slight increase (Figure 7A). It is possible that *ARF17* and miR160 might be differentially distributed in blueberry fruit or cells; alternatively, it could also be possible that the expression of *ARF17* could be mainly regulated by other factors, such as competing endogenous RNAs (ceRNAs), transcription factors and epigenetic regulators.

## 3. Discussion

Many studies have implicated the potential roles of miRNAs in fruit development and biosynthesis of anthocyanin [15,16,17]. Blueberries are fleshy berry fruits recognized for various phenolic compounds, especially anthocyanin pigments, which are involved in a wide variety of health benefits. However, it is totally unknown about the miRNAs and their targets involved in blueberry fruit development and maturation. In this study, genome-wide identification of miRNAs and their target genes involved in blueberry fruit ripening were conducted using small RNA and degradome sequencing, and integrative analysis suggested the miRNA-mediated regulatory networks governing blueberry fruit ripening.

### 3.1. miRNAs and Their Targets during Blueberry Fruit Maturation

Numerous fruit development-associated miRNAs have been identified in fruit-producing plants such as apple [19], grape [20,21], citrus [26], peach [23]. In this study, 84 known miRNAs belonging to 28 families and 16 novel miRNAs were identified in blueberry fruit. 41 miRNAs were differentially expressed between white fruit stage and blue fruit stage (Table 2), suggesting that they might be involved in controlling the cellular and developmental processes during fruit maturation in blueberry. By comparing vco-miRNAs and the ones from other fruit-producing plants such as apple [19], grape [20,21], citrus [26], peach [23], tomato [31] and cucurbits [32], it was found that 21 miRNA families are more common across these fruit-producing plants, suggesting that they might play crucial and fundamental roles in fruit development and maturation in diverse plants. However, 7 miRNAs (such as miR2118, miR2603, miR6149, miR6478, miR7122, miR7693 and miR845) were found only in blueberry fruits. Therefore, we speculate that these 7 miRNAs, together with the 16 novel miRNAs, might be species-specific for blueberry fruit maturation.

It has been proposed that plant miRNAs can mediate gene expression mainly via miRNA-guided cleavage of target transcripts [9]. Therefore, the identification of miRNA targets is a key step to reveal the regulatory roles of miRNAs. In this study, 178 targets were identified for 41 known and 7 novel vco-miRNAs (Tables S2 and S3). Increasing studies indicated that miRNAs seemingly prefer targeting transcription factors in plants [33]. Based on GO analysis, the targets might be mainly involved in DNA-dependent transcription, transcription regulation and protein folding during fruit maturation, which provided preliminary clues for further studying the functions of miRNA-target modules. As have been shown in other species [34,35], the targets of the conserved miRNAs in blueberry are clearly conserved. More importantly, some miRNAs were found to have additional or novel targets in blueberry. For example, miR396s are well-known to target *GRF* family in many plant species [36]. In this study, 33 targets were detected for miR396s in blueberry. Additionally, 8 targets were detected for the 7 novel miRNAs, which are involved in various biological processes. We speculate that these new target genes and the novel miRNAs are likely acting in a species-specific manner during blueberry fruit maturation. Also, it was observed that some miRNA-target pairs showed weak correlation between their expressions. It is not surprising since gene expression is spatio-temporally regulated by a series of complicated mechanisms. The best-known mechanism is that miRNA can sequestered by ceRNAs, therefore leading to deregulation of target gene [37]. Overall, these findings will facilitate our understanding of the miRNA-mediated regulatory network during fruit maturation.

### 3.2. miRNA-Mediated Regulation Network during Blueberry Fruit Maturation

It has been well documented that auxin is not only the most important hormone in the regulation of cell expansion, but also essential for triggering the onset of ripening in tomato and grape berry at low levels [38,39]. In blueberry, it was found that IAA concentration was sharply increased between the S4 and S5 stages, and then began to decline as ripening proceeded [6]. However, little is known about auxin signaling and transport in ripe fruits. In this study, 3 conserved miRNA families (miR160, miR393 and miR396) were identified in blueberry fruit. Based on our degradome data, they may target some auxin regulators such as *ARF17*, *ARF18*, *AFB2*, *TIR1* and *ELONGATOR PROTEIN 6* (*ELP6*) (Figure 7). It has been proposed that ARFs serve as transcription factors that regulate the activation or repression of auxin-responsive genes, and their transcriptional activity can be depressed by heterotypic dimerization with AUX/IAA (AUXIN/INDOLEACETIC ACID) proteins [40]. TIR1 and AFB2 act as auxin receptors to facilitate the degradation of AUX/IAA transcriptional repressor protein, therefore releasing ARFs to activate the transcription of auxin-responsive genes [41]. Likewise, ELP6 may be involved in auxin transport through post-transcriptional modulation of PIN protein levels [42]. Our miRNA sequencing data indicated that the accumulations of miR160, miR393 and miR396 were clearly up-regulated as fruit ripens (Table 1), implying that depression of auxin signaling pathway might occur during fruit ripening in blueberry. More importantly, when coordinated expressions of miR160-*ARF18* and miR396-*ELP6* were investigated during fruit development and ripening, it was observed that the accumulations of miR160b and miR396a-5p were increased as blueberry fruit develops and ripens (Figure 7A,D), while *ARF18* and *ELP6* were conversely expressed (Figure 7A,D). These observations suggest that these miRNAs play roles in controlling the expression of auxin responsive genes during fruit development and maturation in blueberry. Since auxin delays the ripening-related processes such as sugar accumulation, and anthocyanin content [43], we propose that miR160, miR393 and mi396 might promote these ripening-related processes via suppressing the auxin signaling pathway. In addition, players that might connect different hormones and their signaling pathways were also detected in the study. For example, miR159 was detected to target *GAMYB*, which is not only responsive to GAs, but also involved in ABA and sucrose biosynthesis [44]; and *NRT1*/*PTR*, transporter for the plant hormones auxin, ABA, and GA [45], was targeted by a less conserved miRNA (miR7693). These results indicate the possible roles of these miRNAs in controlling fruit development and maturation through mediating hormonal crosstalk.

Plant organs require a proper and precise control of cell proliferation, expansion and differentiation to achieve their final size and shape. Increasing evidences have indicated that miRNAs play pivotal roles in regulating cell proliferation and expansion. For example, miR319-targeted *TCPs* can promote cell expansion and repress cell proliferation in *Arabidopsis* [46], while miR396-targeted *CsGRFs* may play important roles in regulating the growth of fruit and leaf in citrus [47]. Likewise, transposon insertional allele of miRNA172 showed large fruit phenotype, whereas over-expression of miRNA172 significantly reduced fruit size in apple [48]. In this study, the conserved interactions (miR172-*AP2*, miR319-*TCP* and miR396-*GFR1*) were also found in blueberry fruit (Figure 6 and Table S2). Our study also revealed a novel interaction, miR396-*CYP1* (Figure 7 and Table S2), which is very interesting since *CYP1* in tomato (*LeCYP1*) has been reported to be involved in regulation of early fruit development growth [49,50]. Among the 6 stages of fruit development and ripening, the accumulation of all the 3 miRNAs were the highest at blue fruit stage (Table 1 and Figure 7B–D), while their corresponding targets (*AP2*, *TCP*, *GRF1* and *CYP1*) were significantly decreased from onset of fruit ripening (white fruit stage), and reached their minimal level at blue fruit stage (Figure 7B–D). The negative correlation between the expression patterns of these miRNAs and their targets are consistent with a scenario that cell enlargement of blueberry fruit can last until the early stage of fruit maturation. However, the expression patterns between these miRNAs and their targets were not exactly negatively correlated with each other. We speculated that some factors other than miRNAs might also be involved in the regulation of these genes such as transcription factors and epigenetic regulators. Recently, some evidences indicated that miRNAs exquisitely exert dual roles in the regulation of fruit development and maturation. For example, miRNA172 can be involved in fruit growth through negatively modulating cell division and expansion [48], while miR172-targeted *SlAP2a* is a negative regulator of ethylene production during ripening in tomato [51], suggesting that the interaction of miR172-*AP2* might depress fruit development and promote fruit maturation. In this study, *AP2*, *TCP*, *GRF* and *CYP1* were significantly depressed from the onset of fruit maturation until ripening (Figure 7B–D), implying that these genes can promote fruit growth, but delay fruit maturation. We propose that miRNAs possibly act as crucial regulators to balance the expression of these genes, therefore satisfying the requirement of fruit development and maturation.

Many complex molecular changes occur within fruit during maturation. Protein ubiquitination leads to diverse fates of the target proteins, such as protein degradation, alteration in protein trafficking [52]. We identified two ubiquitination-related interactions such as miR396-*UCTH24* (*UBIQUITIN CARBOXYL-TERMINAL HYDROLASE 24*), miR6478-*UCTH9* and miR399-*UBC24* (Figure 6 and Table S2). It has been reported that *UCTHs* play important roles in different cellular processes including cell growth and differentiation through regulating cellular ubiquitin levels [53], while *UBC24* is directly involved in the degradation of ubiquitinated proteins [54]. Differential accumulation of these miRNAs indicated that miRNAs might be involved in the regulation of protein degradation via ubiquitination-related pathway during fruit maturation. Meanwhile, some interactions associated with RNA processing events were identified such as miR403-*AGO2*, miR845-*RBP2I* (*RNA POLYMERASE II SECOND LARGEST SUBUNIT*) and miR7122-*PPR* (Figure 6 and Table S2). Notably, *AGO2*, which was found to be targeted by miR403 in several plant species, is associated with small RNA function [55,56], while PPR proteins are known to mediate specific RNA processing events including RNA editing, transcript processing and translation initiation [57]. These results suggest that diverse events might occur, at both protein and RNA levels, in order to meet the requirement of fruit maturation in blueberry.

Cell wall structure is associated with fruit softening during maturation, and the biochemical characterization of cell wall polymers and their progressive modification during ripening has been documented in blueberry fruits [58]. In this study, two miRNAs (miR166 and miR894) appear to be associated with the modification and formation of cell wall structure (Figure 6 and Table S2). Generally, miR166 can be involved in many developmental processes including seed maturation, leaf polarity determination and secondary wall biosynthesis, via targeting *HD-ZIP III* family genes [14,59,60]. During blueberry fruit maturation, however, miR166 was detected to target *LEUCINE-RICH REPEAT EXTENSIN-LIKE PROTEIN* (*LRX*) (Figure 6 and Table S2), which might be involved in either the regulation of enzymatic activities in the cell wall or the recruitment of enzymes to the appropriate location in the cell wall, therefore altering the structure of cell wall [61]. Meanwhile, *CAFFEOYL SHIKIMATE ESTERASE* (*CSE*) targeted by miR894 (Figure 6 and Table S2), serves as an enzyme central to the biosynthetic pathway of lignin [62], which is a major component of plant secondary cell walls. These findings might be useful for guiding future work to understand the control of fruit firmness and softening.

Fruit coloration during ripening can be achieved by chlorophyll breakdown and production of color metabolites such as anthocyanins and flavonoids. In this study, several miRNA-target pairs appear to be involved in the regulation of fruit coloration in blueberry. For example, miR156, a highly conserved miRNA family, is implicated in the regulation of anthocyanin biosynthesis and fruit development by targeting the *SPL* family genes [63]. In this study, 11 miR156/157 species were identified in blueberry fruit, and most of them showed up-regulated accumulation patterns during fruit ripening (Table 1). As was shown before, *SPL* family was found to be target of miR156/157 in blueberry fruit (Table S3). The accumulation pattern of miR156s during fruit ripening is consistent with the requirement of fruit coloration in blueberry. Furthermore, miR396 and miR_n10 were detected to target *FtsZs* and chloroplastic *BAG1*, respectively (Table S3). *FtsZs* are a class of key players in chloroplast division. It has been reported that the expression of *FtsZ* in fruit was consistent with the chlorophyll concentration in tomato *hp3* mutant [64]. Chloroplastic *BAG1* regulates diverse cellular pathways in chloroplast, such as programmed cell death and stress responses [65]. Therefore, the up-regulation of miR396 and miR_n10 during fruit ripening led us to speculate that these miRNA-target modules might be involved in the regulation of fruit maturation via depression of chloroplast division and/or maintenance, therefore facilitating coloration in blueberry.

**Figure 8:**
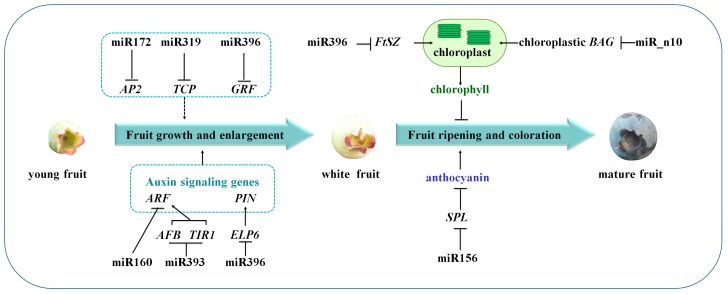
A schematic model showing the proposed roles of miRNAs involved in blueberry fruit development and ripening. T bar and arrow refer to negative and positive effect on downstream effector or biological process, respectively.

In conclusion, numerous miRNAs and their targets during blueberry fruit maturation were identified by next-generation sequencing approach in the study. The interplays between these miRNAs and their targets depict a picture of the regulatory network involved in blueberry fruit development and maturation, and some miRNAs might play vital roles in fruit maturation and coloration via repressing the expression of auxin-responsive genes or the ones responsible for fruit growth and enlargement (Figure 8). These findings will facilitate future investigation of miRNA-mediated mechanisms underlying blueberry fruit development and maturation.

## 4. Materials and Methods

### 4.1. Plant Materials and RNA Extraction

5-year-old blueberry trees (*Vaccinium corymbosum* “Northland”) from clonal propagation grown at the experimental station in Jilin University (Changchun, China) were used. Fruit samples were randomly collected and mixed from 5 or 6 different plants at 5 developmental stages (green pad, green cup, white, pink and blue fruit), which correspond to the stage S3, S4, S5, S6, S7/8 as described previously [6], respectively. The collected samples were immediately frozen in liquid nitrogen and stored at −80 °C.

### 4.2. Construction and Sequencing of sRNA and Degradome Libraries

Total RNAs were extracted from all the fruit samples using RNAprep Pure Plant Kit (Tiangen Inc., Beijing, China) according to the manufacturer’s instructions. Two small RNA (sRNA) libraries and two degradome libraries were constructed from fruits at white stage (WF) and blue stage (BF). Finally, both sRNA and degradome were profiled by single-end sequencing (36 bp) on an Illumina Hiseq 2500. The raw data from the small RNA and degradome sequencing are available at NCBI Short Read Archive (WF-miRNAs, SRX2658700; BF-miRNAs, SRX2676839; WF-degradome, SRX2660622; BF-degradome, SRX2683796).

### 4.3. Analysis of Small RNA Sequencing Data

The raw data were processed to eliminate low quality reads, adapter sequences, junk reads, low complexity sequences and redundant repeats to obtain clean reads Subsequently, the clean reads were subjected to a blast search against Rfam (http://www.sanger.ac.uk/Software/Rfam) and GenBank (http://www.ncbi.nlm.nih.gov/GenBank/) databases to eliminate coding RNAs and Rfam including rRNAs, tRNAs, small nuclear RNAs (snRNAs) and small nucleolar RNAs (snoRNAs). The remaining sequences with length in 18–25 nt were aligned with all the known plant miRNA sequences from miRBase 21.0 (http://www.mirbase.org) to identify candidate miRNAs. Length variation at both 3′ and 5′ ends and one mismatch inside of the sequence were allowed in the alignment. Furthermore, the candidates were mapped to the reference sequences (cranberry genome, the EST sequences and transcriptome sequences of blueberry) to obtain their precursor sequences using the SOAP2 program [66]. To improve the reliability of miRNA identification, the precursors of the identified miRNAs need to satisfy typical secondary hairpin structure as described previously [28]. Novel miRNAs were identified based on the following criteria: (1) mature miRNAs are present in one arm of the hairpin structures, (2) unpaired residues should be no more than 2 between miRNA and miRNA*, (3) hairpin precursors lack internal loop and bulge, (4) secondary structures of the hairpins are steady with high minimal folding free energy index (MFEI) [28]. The hairpin RNA structures were assessed using the Mfold web server (http://unafold.rna.albany.edu/?q=mfold) with the default parameters.

### 4.4. Phylogenetic Analysis of Known miRNAs and Their Precursors in Blueberry

Sequences of the known miRNAs and their precursors in blueberry and grape (*Vitis vinifera*) were collected from sequencing data and miRBase 21.0, and then aligned by ClustalW (Version 2.1, http://www.clustal.org). Subsequently, phylogenetic trees were constructed by maximum likelihood method using software MEGA 7 [67] with 1000 replicates of bootstrap value, and visualized by the online Evolview program (http://www.evolgenius.info/evolview/).

### 4.5. Differential Expression Analysis of miRNAs Between WF and BF Libraries

The expression abundance of miRNAs in two libraries was normalized and if the normalized read counts of miRNAs were less than 1 in both two libraries, and then they were removed. The heatmap was generated using the software HemI1.0 [68]. Furthermore, the differential expression of miRNAs between the two libraries was calculated as: Fold change = miRNA normalized reads in BF/miRNA normalized reads in WF. The miRNAs with fold change greater than 2 or less than 0.5, along with *p*-value ≤ 0.05, were considered as up-regulated or down-regulated ones during maturation, respectively.

### 4.6. Analysis of Degradome Sequencing Data

Degradome analysis and identification of the sliced miRNA targets were performed according to the CleaveLand 3.0 [69]. In brief, the degradome sequences, free from adapter sequences, were mapped to the blueberry transcriptome sequences and ESTs (expressed sequence tags) to generate a degradome density file. Meanwhile, target prediction was performed using TargetFinder program. The alignment between miRNA and mRNA (blueberry transcriptome sequences and ESTs) were generated using ‘targetfinder.pl’, and the parameters were set as default. Thereafter, the degradome density file and target predictions were compared and significant hits were identified as target plots (t-plots) [26]. The potential target transcripts were classified into five main categories based on the abundance of degradome reads as reported previously [26]. Gene Ontology (GO) annotations were assigned using the Gene Ontology tool (http://www.Geneontology.org/).

### 4.7. qRT-PCR Validation of miRNAs and Their Potential Targets from Degradome Data

Total RNA was isolated from fruit samples at 5 developmental stages (green pad, green cup, white, pink and blue fruit) using RNAPlant Plus Reagent kit (Tiangen Inc., Beijing, China) according to the manufacturer’s instruction. Subsequently, polyethylene glycol (PEG) 8000 was used to precipitate of high-molecular-weight (HMW) RNAs. Finally, small RNAs could be easily recovered from supernatant by ethanol precipitation [70]. Reverse transcription was performed with PrimeScript™ RT reagent Kit with gDNA Eraser (Takara Inc., Dalian, China) using specific stem-loop RT primers for miRNAs and the oligo dT primer for targets (Table S4). qRT-PCR was subsequently conducted with an ABI StepOnePlus PCR system and SYBR Premix Ex Taq (Takara Inc.), using *ACTIN* (CF811156) and *U6* as internal control for targets and miRNAs, respectively. Three biological replicates with three technical replicates for each biological replicate were performed for each sample, and data were analyzed by the software ABI StepOnePlus v2.3 (Applied Biosystems, Foster City, CA, USA). The stem-loop primers and target qRT-PCR primers are listed in Tables S4 and S5, respectively.

### 4.8. RNA Ligase-Mediated 5′ RACE

RNA ligase-mediated rapid amplification of 5′ cDNA ends (RLM-5′ RACE) was performed with SMARTer™ RACE cDNA Amplification Kit (Clontech Laboratories Inc., PaloAlto, CA, USA) according to the manufacturer’s instructions. Briefly, RNA adapter was ligated to the purified RNAs. The ligation products were reverse transcribed, followed by nested PCR amplifications using universal primers and gene specific primers (Table S6). The RACE products were gel purified, cloned and sequenced.

## Figures and Tables

**Table 1:** Characteristics of total miRNAs identified from BF (blue fruit) and WF (white fruit) libraries in blueberry.

ID	miRNA Sequences (5′-3′)	LT ^1^	TP ^2^	Accession ^3^	Str ^4^	Start	End	HOMO ^5^/NM ^6^	MFEI ^7^	FPKM ^8^	
										BF	WF
vco-miR156a	CUGACAGAAGAGAGUGAGCAC	21	5′	JOTO01194981	−	104	229	gma-miR156a	1	9.57	5.41
vco-miR156b-5p	UUGACAGAAGAGAGUGAGCAC	21	5′	JOTO01158632	+	1177	1373	mtr-miR156b-5p	0.9	2.50	2.40
vco-miR156c-3p	GCUCACUUUUCUUCUGUCAUU	21	3′	JOTO01174941	−	2100	2189	mdm-miR156w-p3	1.3	9.16	0.00
vco-miR156c-5p	CUGACAGAAGAGAGUGAGCAC	21	5′	JOTO01174941	−	2100	2189	gma-miR156a	1.3	9.57	5.41
vco-miR156d-3p	GCUCUCUAUGCUUCUGUCAUC	21	3′	JOTO01177646	+	179	337	stu-miR156d-	0.8	58.27	36.64
vco-miR156e-3p	GCUCUCUAUGCUUCUGUCAUC	21	3′	JOTO01177646	−	1	159	mtr-miR156h-3p	0.8	58.27	36.64
vco-miR156e-5p	UUGACAGAAGAUAGAGAGCACUG	23	5′	JOTO01177646	−	1	159	mtr-miR156e	0.8	1.66	6.01
vco-miR156f	GCUCUCUAGUCUUCUGUCAUC	21	3′	JOTO01152293	+	62	166	gma-miR156e-p3	1.1	10.82	0.00
vco-miR156g-5p	UGACAGAAGAGAGUGAGCAC	20	5′	JOTO01184662	+	3109	3203	mtr-miR156b-5p	1	32.46	4.81
vco-miR156h-3p	GCUCACUUCUCUCUCUGUCAUC	22	3′	JOTO01167006	+	3494	3585	mtr-miR156i-3p	1.2	23.31	3.60
vco-miR157a	UUGACAGAAGAUAGAGAGCAC	21	5′	JOTO01152293	+	65	163	gra-miR157a	1.1	11.65	39.64
vco-miR159a	GCGGCGACGGGGGCGGUU	18	3′	JOTO01109152	−	4086	4230	zma-miR159e-p3	0.6	3.75	0.00
vco-miR160a-5p	UGCCUGGCUCCCUGUAUGCA	20	5′	JOTO01191503	+	4712	4793	ath-miR160a-5p	1.1	7.49	0.00
vco-miR160b	UGCCUGGCUCCCUGAAUGCCAU	22	5′	JOTO01197084	−	3443	3530	mes-miR160e	1.1	1.66	1.20
vco-miR162a-3p	UCGAUAAACCUCUGCAUCCAG	21	3′	JOTO01180996	+	1229	1340	mtr-miR162	0.8	414.55	432.47
vco-miR162a-5p	UGGACGCAGCGGUUCAUCGAUC	22	5′	JOTO01180996	+	1229	1340	ath-miR162a-5p	0.8	0.83	1.20
vco-miR164a	AUGGAGAAGCAGGGCACGUGU	21	5′	JOTO01183560	−	3074	3198	mtr-miR164a	1	4.99	6.01
vco-miR166a	UCGGACCAGGCUUCAUUCCCCU	22	3′	JOTO01182330	−	3021	3176	mtr-miR166a	0.9	89.49	30.03
vco-miR166b-3p	UCGGACCAGGCUUCAUUCCCC	21	3′	JOTO01175424	−	3267	3364	ptc-miR166a	1	1377.66	980.27
vco-miR166b-5p	GGAAUGCUGUCUGGUUCGAGG	21	5′	JOTO01175424	−	3267	3364	ptc-miR166g-p5	1	77.42	1.20
vco-miR166c-3p	UGUCGGACCAGGCUUCAUUCC	21	3′	JOTO01191740	−	2020	2122	mtr-miR166a	1.2	10.82	11.41
vco-miR166c-5p	AAUGUUGUCUGGUUCGAGAUC	21	5′	JOTO01191740	−	2020	2122	mdm-miR166c-p5	1.2	1.66	2.40
vco-miR166d-3p	UCGGACCAGGCUUCAUUCCUC	21	3′	JOTO01177553	+	1331	1462	mtr-miR166c	0.9	469.90	853.53
vco-miR166d-5p	GGGAUGUUGUCUGGCUCGAUG	21	5′	JOTO01177553	+	1331	1462	sly-miR166c-5p	0.9	318.82	461.30
vco-miR166e-3p	UCUCGGACCAGGCUUCAUUCU	21	3′	JOTO01175424	−	3207	3307	cme-miR166i-p3	1	159.83	72.08
vco-miR166e-5p	GGAAUGCUGUCUGGCUCGAGG	21	5′	JOTO01175424	−	3207	3307	mtr-miR166g-5p	1	0.00	2.40
vco-miR166f-5p	GGAAUGUUGUCUGGCUCGAGG	21	5′	JOTO01193807	+	4411	4505	mtr-miR166g-5p	1	469.49	353.19
vco-miR167a	UGAAGCUGCCAGCAUGAUCUAA	22	5′	JOTO01143215	−	81	168	mdm-miR167b	0.9	0.83	2.40
vco-miR167b	UGAAGCUGCCAGCAUGAUCUGA	22	5′	JOTO01169118	−	703	820	mtr-miR167b-5p	1	21.64	21.62
vco-miR171a-3p	UUGAGCCGCGCCAAUAUCACU	21	3′	JOTO01145355	−	1932	2031	vvi-miR171f	1.1	39.12	0.00
vco-miR171a-5p	CGAUGUUGGUGAGGUUCAAUC	21	5′	JOTO01145355	−	1932	2031	mtr-miR171e-5p	1.1	7.49	0.00
vco-miR171b	AAGGGUGUUGUUCGAUUAA	19	5′	JOTO01109152	−	2162	2277	ath-miR171b-p5	0.6	6.66	0.00
vco-miR172a-3p	AGAAUCUUGAUGAUGCUGCAU	21	3′	JOTO01171463	+	458	595	mtr-miR172b	1.1	8.32	1.20
vco-miR172a-5p	GCGGCAUUAUCAAGAUUCAC	20	5′	JOTO01171463	+	458	595	gra-miR172a	1.1	64.10	7.21
vco-miR319a	UGGACUGAAGGGAGCUCCCUU	21	5′	JOTO01138414	−	1309	1470	mtr-miR319a-3p	0.7	365.02	49.85
vco-miR319b	UUGGACUGAAGGGAGCUCCC	20	3′	JOTO01184324	+	5377	5438	mtr-miR319a-3p	0.7	262.63	15.62
vco-miR319c	UGGACUGAAGGGAGCUCCCUU	21	3′	JOTO01169050	+	2402	2535	mtr-miR319a-3p	0.6	365.02	49.85
vco-miR390a-3p	CGCUAUCCAUCCUGAGUUUCA	21	3′	JOTO01159657	−	2355	2501	gma-miR390a-3p	1	4.58	1.80
vco-miR390a-5p	AAGCUCAGGAGGGAUAGCGCC	21	5′	JOTO01159657	−	2355	2501	mtr-miR390	1	1.25	1.20
vco-miR390b-3p	CGCUAUCCAUCCUGAGUUUCA	21	3′	JOTO01180795	+	4526	4630	gma-miR390	1	4.58	1.80
vco-miR390b-5p	AAGCUCAGGAGGGAUAGCGCC	21	5′	JOTO01180795	+	4526	4630	mtr-miR390	1	1.25	1.20
vco-miR393a-3p	AUCAUGCGAUCCCUUAGGAUU	21	3′	JOTO01199451	+	28	171	ptc-miR393a-3p	1	2.91	0.60
vco-miR393a-5p	UCCAAAGGGAUCGCAUUGAU	20	5′	JOTO01199451	+	28	171	mtr-miR393a	1	2.50	1.20
vco-miR393b-3p	AUCAUGCGAUCCCUUAGGAUU	21	3′	JOTO01199451	+	2549	2697	ptc-miR393a-3p	1	2.91	0.60
vco-miR393b-5p	UCCAAAGGGAUCGCAUUGAU	20	5′	JOTO01199451	+	2549	2697	mtr-miR393a	1	2.50	1.20
vco-miR394a-5p	UUGGCAUUCUGUCCACCUCC	20	5′	JOTO01174460	+	2780	2935	gma-miR394a-5p	0.8	87.40	0.00
vco-miR395a	CUGAAGUGUUUGGGGGAACUC	21	3′	JOTO01195534	−	9719	9799	vvi-miR395a	1	70.76	82.89
vco-miR396a-3p	GUUCAAGAAAUCUGUGGGAGA	21	3′	JOTO01179423	+	3728	3868	mtr-miR396a-3p	1.3	4.16	6.01
vco-miR396a-5p	UUCCACAGCUUUCUUGAACUU	21	5′	JOTO01179423	+	3728	3868	mtr-miR396a-5p	1.3	432.86	151.97
vco-miR396b-3p	GUUCAAUAAAGCUGUGGGAAG	21	3′	JOTO01198645	+	118	264	mtr-miR396b-3p	1.1	60.77	34.24
vco-miR396b-5p	UUCCACAGCUUUCUUGAACU	20	5′	JOTO01198645	+	118	264	mtr-miR396b-5p	1.1	69.51	22.82
vco-miR396c-3p	GUUCGAGAAAGCUGUGGGAAG	21	3′	JOTO01196729	−	5681	5840	mtr-miR396b-3p	0.9	3.33	2.40
vco-miR396c-5p	UUCCACAGCUUUCUUGAACUC	21	5′	JOTO01196729	−	5681	5840	mtr-miR396a-5p	0.9	165.24	34.24
vco-miR396d-3p	GUUCAAUUAAGCUGUGGGAAG	21	3′	JOTO01198645	−	421	567	mtr-miR396b-3p	1.1	26.64	9.61
vco-miR396d-5p	UUCCACAGCUUUCUUGAACUC	21	5′	JOTO01198645	−	421	567	mtr-miR396b-5p	1.1	165.24	34.24
vco-miR396e-3p	GUUCACUAAAGCUGUGGAAAG	21	3′	JOTO01155840	+	2054	2198	mtr-miR396b-3p	1	27.47	21.62
vco-miR396e-5p	UUCCACAGCUUUCUUGAACUG	21	5′	JOTO01155840	+	2054	2198	mtr-miR396b-5p	1	124.45	16.22
vco-miR396f-3p	GUUCAAUAAAGCUGUGGGAAG	21	3′	JOTO01198645	+	8127	8264	mtr-miR396b-3p	1.1	60.77	34.24
vco-miR396f-5p	UUCCACAGCUUUCUUGAACUG	21	5′	JOTO01198645	+	8127	8264	mtr-miR396b-5p	1.1	124.45	16.22
vco-miR396g	GCUCAAGAAAGCUGUGGGAAG	21	5′	JOTO01192216	+	28	102	mtr-miR396b-3p	0.6	3.33	14.42
vco-miR398a-3p	UGUGUUCUCAGGUCACCCCUU	21	3′	JOTO01181367	−	2816	2942	mtr-miR398a-3p	0.8	4.16	0.00
vco-miR398a-5p	GGAGUGACACUUAGAACACACG	22	5′	JOTO01181367	−	2816	2942	mtr-miR398a-5p	0.8	0.42	1.20
vco-miR398b-3p	UGUGUUCUCAGGUCACCCCUU	21	3′	JOTO01178370	−	4047	4163	mtr-miR398a-3p	1.1	4.16	0.00
vco-miR398b-5p	GGAGUGACACUGAGAACACU	20	5′	JOTO01178370	−	4047	4163	mtr-miR398a-5p	1.1	11.24	0.00
vco-miR398c-3p	UGUGUUCUCAGGUCGCCCCUG	21	3′	JOTO01177986	−	4722	4858	mtr-miR398b	1	17.48	12.01
vco-miR398c-5p	GGAGCGACCUGAGAUCACAUG	21	5′	JOTO01177986	−	4722	4858	ptc-miR398c-5p	1	69.09	22.82
vco-miR399a	UGCCAAAGGAGAAUUGCCCUG	21	3′	JOTO01171567	−	2276	2397	mtr-miR399c	1	4.99	7.21
vco-miR399b	UGCCAAAGGAGAGUUGCCCUG	21	3′	JOTO01187770	−	6181	6281	mtr-miR399l	1	1.66	10.81
vco-miR403a	UUAGAUUCACGCACAAACUCUG	22	3′	JOTO01197485	−	10,488	10,629	gma-miR403a	1.1	89.90	34.84
vco-miR535a-3p	GUGCUCCCUAUCGUCGUCAU	20	3′	JOTO01190921	+	974	1058	mes-miR535a-p3	1	276.36	241.46
vco-miR535a-5p	UGACGACGAGAGAGAGCACGC	21	5′	JOTO01190921	+	974	1058	mdm-miR535d	1	17.06	66.07
vco-miR535b	UGACGACGAGAGAGAGCACGC	21	5′	JOTO01171383	+	217	311	mdm-miR535d	1.1	17.06	66.07
vco-miR845	UGCUCUGAUACCAAUUGUUGA	21	5′	JOTO01134176	−	526	624	bdi-miR845	0.9	3.33	4.81
vco-miR894	UUCACGUCGGGUUCACCA	18	5′	JOTO01175668	+	4734	4832	ppt-miR894	0.5	340.46	1055.95
vco-miR2111	ACCGGGUAAUCUGCAUCCUGA	21	5′	JOTO01195655	−	6181	6282	tcc-miR2111-p5	1.1	0.83	1.20
vco-miR2118	UUACCGACUCCACCCAUACCUA	22	3′	JOTO01101378	−	99	199	mtr-miR2118	1.3	26.64	20.42
vco-miR2603	GGUCCCUGCCCUUUGUACA	19	5′	JOTO01109152	−	4086	4230	mtr-miR2603	0.6	4.58	1.20
vco-miR6149a	CUUCAUACGCACCUGAAUCGG	21	5′	JOTO01189719	+	7347	7491	nta-miR6149a	1	14.15	6.01
vco-miR6478	UCGACCUUAGCUCAGUUGGUA	21	5′	JOTO01186167	−	6643	6834	ptc-miR6478	0.6	201.03	330.36
vco-miR7122a-3p	ACCGUGUUUCUCUGUAUAAAU	21	3′	JOTO01165247	+	1957	2063	mdm-miR7122a-p3	1.1	748.35	595.85
vco-miR7122a-5p	UUAUACAGAAAAAUCACGGUCG	22	5′	JOTO01165247	+	1957	2063	mdm-miR7122a	1.1	2.08	0.00
vco-miR7122b-3p	ACCGUGUUUCUCUGUAUAAAU	21	3′	JOTO01194243	−	8897	9055	mdm-miR7122a-p3	1	748.35	595.85
vco-miR7122b-5p	UUAUACAGAAAAAUCACGGUCG	22	5′	JOTO01194243	−	8897	9055	mdm-miR7122a	1	2.08	0.00
vco-miR7693-5p	GCAUCGAUGAAGAGCGUA	18	5′	JOTO01109152	−	3655	3758	osa-miR7693-p5	0.5	8.32	0.60
vco-miR_n01	UCCGAUCUGAAUUCAAGCGAA	21	3′	JOTO01190684	+	1726	1822	1	1.4	6.66	10.21
vco-miR_n02	UCCGAUCUGAAUUCAAGCGAA	21	3′	JOTO01190684	−	1689	1787	0	1.4	6.66	10.21
vco-miR_n03	GUGUGGCUGGGAGUGGCUCCU	21	3′	JOTO0118763	−	468	619	0	1.6	11.65	18.02
vco-miR_n04	GUGUGGCUGGGAGUGGCUCCC	21	3′	JOTO0118763	+	541	703	1	1.5	11.65	29.43
vco-miR_n05	ACACAUCUGUGUCUGAAUCUGUGC	24	3′	JOTO01183568	+	4231	4401	1	1.3	633.47	1071.57
vco-miR_n06	CCGAUAUGAGUCUAACAUGCC	21	3′	JOTO01181717	−	2207	2389	1	1.7	21.23	10.21
vco-miR_n07	CCCGUUGGAUGCAUUUUCU	19	3′	JOTO01180185	+	2426	2591	1	1.5	69.92	16.82
vco-miR_n08-5p	UGAGUUGUAAGCAAAGUCCGC	21	5′	JOTO01179705	−	4366	4501	2	1.2	26.22	18.02
vco-miR_n08-3p	GGCGGACUUUGCUUAGAACCC	21	3′	JOTO01179705	−	4366	4501	2	1.2	198.95	201.82
vco-miR_n09	UGGACGAUCGGGAUGUGCUAGUGU	24	3′	JOTO01178111	−	3319	3476	0	1.4	19.56	13.21
vco-miR_n10	GUGCUUUCUAUCGUCGUCAU	20	3′	JOTO01171383	+	217	311	2	1.1	350.45	97.31
vco-miR_n11	CCCGUUGGAUGCAUUUUCUGA	21	3′	JOTO01167166	−	2168	2315	1	1.1	116.12	21.02
vco-miR_n12-5p	CCUCCGACUCUAUAGACGAAG	21	5′	JOTO01135109	−	1462	1683	1	1.5	13.32	52.86
vco-miR_n12-3p	UCGUAUAUAGAGUCGGAGGCU	21	3′	JOTO01135109	−	1462	1683	1	1.5	11.65	58.86
vco-miR_n13	GUUGAUCUCGGAUCGGACGG	20	5′	JOTO01100636	+	836	981	0	1.3	10.41	11.41
vco-miR_n14	CGTCCGAUCCAAGAUCAACGGUCU	24	3′	JOTO01100636	−	731	878	1	1.3	19.15	4.81

^1^ the length of miRNA; ^2^ location of mature miRNAs on secondary stem-loop structures of pre-miRNA sequences; ^3^ Accession number of scaffold containing pre-miRNA; ^4^ sense or nonsense strand; ^5^ HOMO and ^6^ NM refer to closely-related homolog and number of mismatched nucleotide between novel miRNA and miRNA*, respectively; ^7^ MFEI and ^8^ FPKM indicate minimal folding free energy index and fragments per kilobase of transcript per million fragments mapped, respectively.

**Table 2:** Differentially expressed miRNAs between WF (white fruit) and BF (blue fruit) libraries in blueberry.

miRNAs	BF (norm)	WF (norm)	Fold Change	log_2_(Fold Change) ^1^	*p*-Value (Fisher Test)	Sig-Lable ^2^	Up/Down
vco-miR156c-3p	9.16	0.00	inf	inf	1.29 × 10^−5^	**	up
vco-miR156f	10.82	0.00	inf	inf	1.06 × 10^−6^	**	up
vco-miR159a	3.75	0.00	inf	inf	6.72 × 10^−3^	**	up
vco-miR160a-5p	7.49	0.00	inf	inf	1.58×10^-4^	**	up
vco-miR171a-3p	39.12	0.00	inf	inf	6.54 × 10^−22^	**	up
vco-miR171a-5p	7.49	0.00	inf	inf	1.58 × 10^−4^	**	up
vco-miR171b	6.66	0.00	inf	inf	1.58 × 10^−4^	**	up
vco-miR394a-5p	87.40	0.00	inf	inf	5.47 × 10^−48^	**	up
vco-miR398a-3p	4.16	0.00	inf	inf	6.72 × 10^−3^	**	up
vco-miR398b-3p	4.16	0.00	inf	inf	6.72 × 10^−3^	**	up
vco-miR398b-5p	11.24	0.00	inf	inf	1.06 × 10^−6^	**	up
vco-miR166b-5p	77.42	1.20	64.44	6.01	8.31 × 10^−41^	**	up
vco-miR319b	262.63	15.62	16.82	4.07	2.33 × 10^−120^	**	up
vco-miR7693-5p	8.32	0.60	13.86	3.79	3.03 × 10^−4^	**	up
vco-miR172a-5p	64.10	7.21	8.89	3.15	2.25 × 10^−27^	**	up
vco-miR396e-5p	124.45	16.22	7.67	2.94	8.80 × 10^−50^	**	up
vco-miR396f-5p	124.45	16.22	7.67	2.94	8.80 × 10^−50^	**	up
vco-miR319a	365.02	49.85	7.32	2.87	2.54 × 10^−141^	**	up
vco-miR319c	365.02	49.85	7.32	2.87	2.54 × 10^−141^	**	up
vco-miR172a-3p	8.32	1.20	6.93	2.79	3.03 × 10^−4^	**	up
vco-miR156h-3p	23.31	3.60	6.47	2.69	1.57 × 10^−9^	**	up
vco-miR396c-5p	165.24	34.24	4.83	2.27	6.37 × 10^−57^	**	up
vco-miR396d-5p	165.24	34.24	4.83	2.27	6.37 × 10^−57^	**	up
vco-miR2603	4.58	1.20	3.81	1.93	8.79 × 10^−3^	**	up
vco-miR396b-5p	69.51	22.82	3.05	1.61	1.73 × 10^−20^	**	up
vco-miR398c-5p	69.09	22.82	3.03	1.60	4.57 × 10^−20^	**	up
vco-miR166a	89.49	30.03	2.98	1.58	2.66 × 10^−25^	**	up
vco-miR396a-5p	432.86	151.97	2.85	1.51	3.86 × 10^−114^	**	up
vco-miR396d-3p	26.64	9.61	2.77	1.47	3.00 × 10^−8^	**	up
vco-miR403a	89.90	34.84	2.58	1.37	1.39 × 10^−23^	**	up
vco-miR390a-3p	4.58	1.80	2.54	1.35	2.35 × 10^−2^	*	up
vco-miR390b-3p	4.58	1.80	2.54	1.35	2.35 × 10^−2^	*	up
vco-miR6149a	14.15	6.01	2.36	1.24	1.51 × 10^−4^	**	up
vco-miR166e-3p	159.83	72.08	2.22	1.15	5.95 × 10^−37^	**	up
vco-miR_n11	116.12	21.02	5.52	2.47	2.51 × 10^−42^	**	up
vco-miR_n07	69.92	16.82	4.16	2.06	1.67 × 10^−23^	**	up
vco-miR_n14	19.15	4.81	3.98	1.99	4.17 × 10^−7^	**	up
vco-miR_n10	350.45	97.31	3.60	1.85	6.93 × 10^−105^	**	up
vco-miR_n06	21.23	10.21	2.08	1.06	7.25 × 10^−6^	**	up
vco-miR894	340.46	1055.95	0.32	−1.63	3.70 × 10^−4^	**	down
vco-miR_n12-3p	11.65	58.86	0.20	−2.34	3.46 × 10^−2^	*	down

^1^ |log_2_(fold change)| ≥ 1; ^2^ Sig-label ** *p*-value ≤ 0.01, * 0.01 < *p*-value ≤ 0.05. inf refers to infinite fold change or log_2_(fold change).

## Supplementary Materials

Supplementary materials can be found at www.mdpi.com/1422-0067/18/12/2767/s1.
