# Supplementary material for: Comparative Analysis of Fruit Ripening-Related miRNAs and Their Targets in Blueberry Using Small RNA and Degradome Sequencing
Source: Int J Mol Sci. 2017 Dec 19;18(12):2767. doi: 10.3390/ijms18122767 (PMC5751366; doi:10.3390/ijms18122767)
Supplement: Supplementary file 1 [file ijms-18-02767-s001.zip › Table S1-2.docx]

**Table S1** Statistical analysis of sequencing reads from small RNA and degradome libraries (BF and WF) in blueberry.

|  | Category | BF^1^ Library | |  | WF^2^ Library | |
| --- | --- | --- | --- | --- | --- | --- |
|  |  | Total reads | Unique reads |  | Total reads | Unique reads |
| Small RNA data | Raw reads | 31,398,541 | 5,994,516 |  | 13,884,034 | 3,080,942 |
|  | Clean reads | 9,561,773 | 2,414,556 |  | 4,582,251 | 1,141,999 |
|  | Valid reads | 7,179,774  (22.87%) | 2,277,307  (37.99%) |  | 3,054,344  (22.00%) | 1,063,932  (34.53%) |
|  | rRNA | 1,795,743  (5.72%) | 98,863  (0.31%) |  | 770,570  (5.55%) | 51,110  (0.37%) |
|  | tRNA | 384,076  (1.22%) | 22,074  (0.07%) |  | 686,636  (4.95%) | 18,698  (0.13%) |
|  | snoRNA | 12,085  (0.04%) | 3,661  (0.01%) |  | 6,844  (0.05%) | 2,182  (0.02%) |
|  | snRNA | 5,682  (0.02%) | 2,528  (0.01%) |  | 3,889  (0.03%) | 1,432  (0.01%) |
|  | other Rfam RNA | 184,413  (0.59%) | 10,123  (0.03%) |  | 59,968  (0.43%) | 4,645  (0.03%) |
| Degradome data | Raw Reads | 13,702,581 | 4,999,617 |  | 10,686,450 | 4,523,764 |
|  | Mappable Reads | 13,629,304  (99.47%) | 4,964,909  (99.31%) |  | 10,631,033  (99.48%) | 4,492,514  (99.31%) |
|  | Transcript Mapped Reads | 8,167,346  (59.60%) | 2,690,920  (53.82%) |  | 5,981,431  (55.97%) | 2,461,509  (54.41%) |
|  | Number of input Transcript | 71,181 |  |  | 71,181 |  |
|  | Number of Covered Transcript | 40,167  (56.43%) |  |  | 40,302  (56.62%) |  |

^1^ Blue fruit, ^2^ White fruit

**Table S2** Identified targets for miRNAs by degradome sequencing in blueberry.

| **miRNAs** | **Transcript** | **Alignment Score** | **Degradome Cleavage Site** | **BF_Degradome Category** | **WF_Degradome Category** | **Gene** |
| --- | --- | --- | --- | --- | --- | --- |
| vco-miR156/157 | comp42469_c0 | 2 | 1270 |  | 0 | Squamosa promoter-binding-like protein 2 |
|  | comp42767_c0 | 2 | 1610 | 0 | 0 | Squamosa promoter binding protein like 6 |
|  | comp14442_c0 | 2 | 267 |  | 0 | Promoter-binding protein SPL9 |
|  | comp44195_c0 | 3.5 | 72 |  | 2 | Auxin-responsive protein IAA27 |
| vco-miR159 | comp51625_c0 | 4 | 709 | 2 |  | Molybdate transporter 2 |
|  | gi354708794 | 4 | 217 |  | 2 | Protein RETICULATA-RELATED 4 |
|  | comp26967_c0 | 4 | 1605 | 2 | 1 | Transcription factor GAMYB |
| vco-miR160 | comp49741_c0 | 1.5 | 1832 |  | 4 | Auxin response factor 18 |
|  | comp13133_c0 | 1.5 | 271 | 0 | 0 | Auxin response factor 17 |
| vco-miR162 | comp47592_c0 | 2 | 3574 | 0 | 0 | CAP-Gly domain-containing linker protein 1 |
| vco-miR164 | comp44771_c0 | 2.5 | 839 |  | 4 | NAC domain-containing protein 100 |
| vco-miR166 | comp45686_c0 | 4 | 969 | 2 |  | Protein S-acyltransferase 8 |
|  | comp51740_c0 | 4 | 1093 |  | 4 | leucine-rich repeat extensin-like protein |
| vco-miR172 | comp39072_c0 | 3 | 1235 | 0 | 0 | Transcription factor APETALA2 |
|  | gi354715779 | 4 | 75 | 2 |  | GTP-binding protein SAR1A-like |
| vco-miR319 | comp36266_c0 | 3.5 | 593 | 4 |  | TCP transcription factor |
| vco-miR393 | comp48356_c0 | 1 | 2187 | 0 | 0 | Auxin signaling f-box 2 |
|  | comp116543_c0 | 1.5 | 1217 | 0 | 0 | Transport inhibitor response 1 |
| vco-miR395 | comp37266_c0 | 1.5 | 445 | 1 | 0 | ATP sulfurylase(APS2) |
| vco-miR396 | comp44163_c0 | 4 | 837 |  | 0 | Growth-regulating factor 1 |
|  | comp50104_c0 | 4 | 442 |  | 2 | Ubiquitin carboxyl-terminal hydrolase 24 |
|  | comp33755_c0 | 3.5 | 465 |  | 2 | Elongator complex protein 6 |
|  | comp43217_c0 | 4 | 1173 | 2 |  | TATA-binding protein-associated factor BTAF1 |
|  | gi354708754 | 4 | 619 |  | 4 | cell division protein FtsZ homolog 2-2 |
|  | gi354713616 | 4 | 81 | 2 | 2 | Cyclophilin 1 |
|  | gi354702167 | 4 | 142 | 2 | 2 | Peptidyl-prolylcis-trans isomerase 1 |
|  | comp41180_c0 | 4 | 791 | 2 | 2 | Nudix hydrolase 2-like |
|  | comp47891_c0 | 4 | 50 | 1 |  | Vicilin-like seed storage protein |
|  | comp46274_c0 | 4 | 126 | 2 |  | Calcium homeostasis endoplasmic reticulum protein-like |
|  | comp51385_c0 | 3 | 39 |  | 2 | Protein IQ-DOMAIN 14 |
|  | gi354705083 | 3.5 | 566 | 2 | 4 | neutral ceramidase-like |
| vco-miR399 | comp49095_c0 | 3.5 | 190 | 2 | 1 | Ubiquitin-conjugating enzyme E2 24 |
| vco-miR403 | comp40107_c0 | 4 | 235 | 0 | 4 | F-box protein PP2-A12 |
|  | comp25090_c0 | 2.5 | 111 | 2 | 2 | protein argonaute 2 |
| vco-miR845 | gi354710052 | 2.5 | 254 |  | 2 | RNA polymerase II second largest subunit |
| vco-miR894 | comp42848_c0 | 4 | 498 | 2 |  | Heat shock 70 kda protein 15-like |
|  | comp49293_c0 | 4 | 1004 |  | 4 | caffeoyl shikimate esterase |
| vco-miR2118 | comp27065_c0 | 3 | 161 | 0 | 0 | Disease resistance RPP13-like protein 1 |
| vco-miR6478 | comp23367_c0 | 4 | 128 |  | 2 | Ubiquitin carboxyl-terminal hydrolase 9 |
| vco-miR_n07 | comp42440_c0 | 3.5 | 599 | 2 |  | Oleosin 1 |
| vco-miR7122 | comp13410_c0 | 3.5 | 129 | 0 | 0 | PREDICTED: Sesamum indicum pentatricopeptide repeat-containing protein At1g63330-like (LOC105172068), mRNA |
| vco-miR7693 | comp47249_c0 | 3.5 | 16 |  | 2 | protein NRT1/ PTR FAMILY 6.4 |
| vco-miR_n08-5p | comp31522_c0 | 4 | 118 | 4 |  | Pre-mrnapolyadenylation factor fip-1-like |
| vco-miR_n09 | comp49514_c0 | 4 | 1469 | 3 |  | UDP-glucose: flavonoid 3-O-glucosyltransferase |
| vco-miR_n10 | comp46611_c0 | 4 | 644 |  | 2 | BAG family molecular chaperone regulator 8 |
| vco-miR_n13 | comp29714_c0 | 4 | 141 | 4 |  | Retrotransposoncopia-like GBRE-1 polyprotein gene |
