# Supplementary material for: Comparative Analysis of Fruit Ripening-Related miRNAs and Their Targets in Blueberry Using Small RNA and Degradome Sequencing
Source: Int J Mol Sci. 2017 Dec 19;18(12):2767. doi: 10.3390/ijms18122767 (PMC5751366; doi:10.3390/ijms18122767)
Supplement: Supplementary file 1 [file ijms-18-02767-s001.zip › Figure S3.pdf]

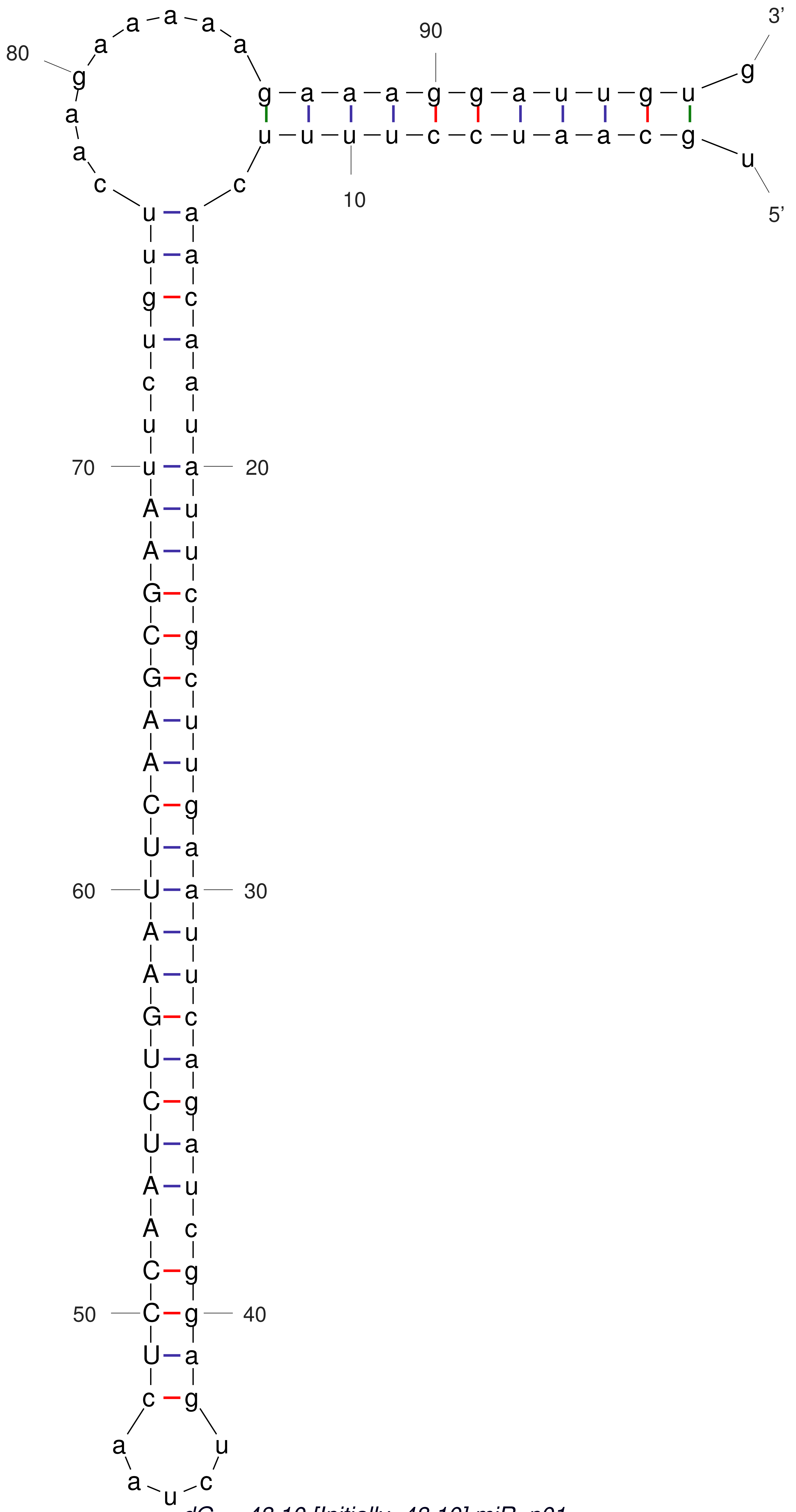

dG = -48.10 [Initially -48.10] miR_n01

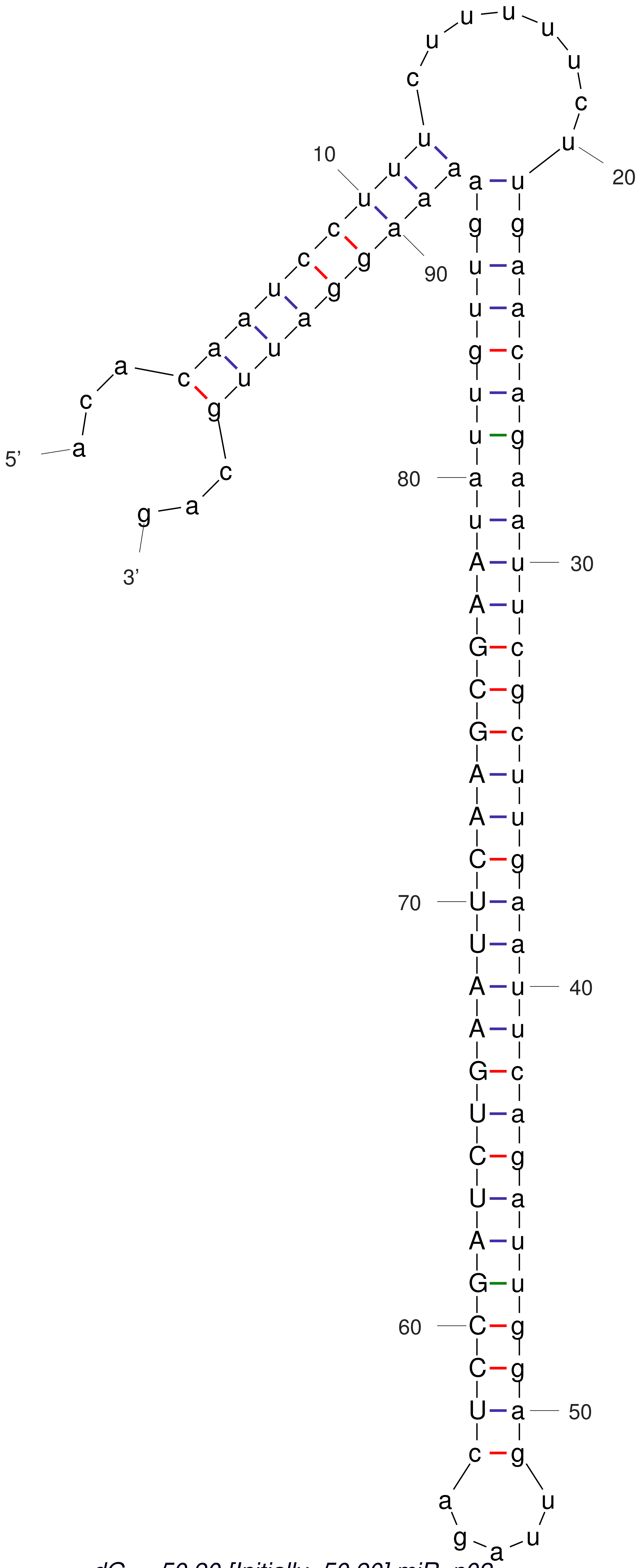

dG = -50.90 [Initially -50.90] miR_n02

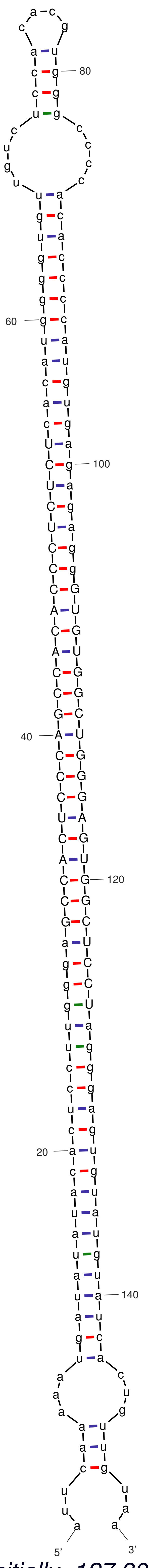

dG = -127.80 [Initially -127.80] miR_n03

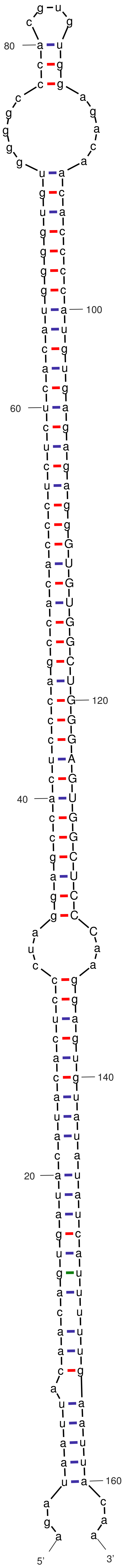

dG = -121.00 [Initially -121.00] miR_n04

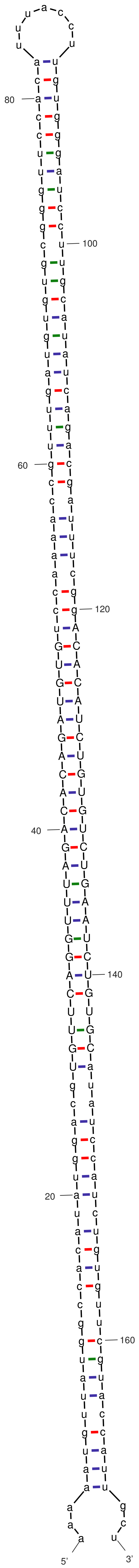

dG = -100.60 [Initially -100.60] miR_n05

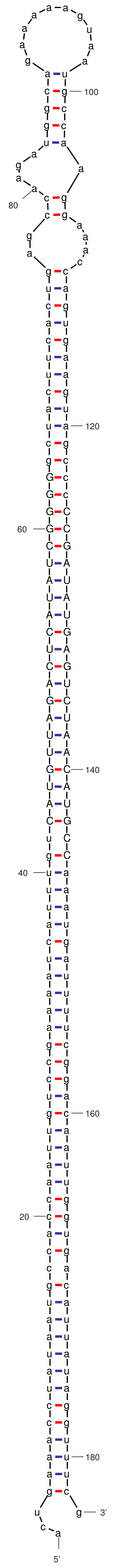

dG = -131.50 [Initially -131.50] miR_n06

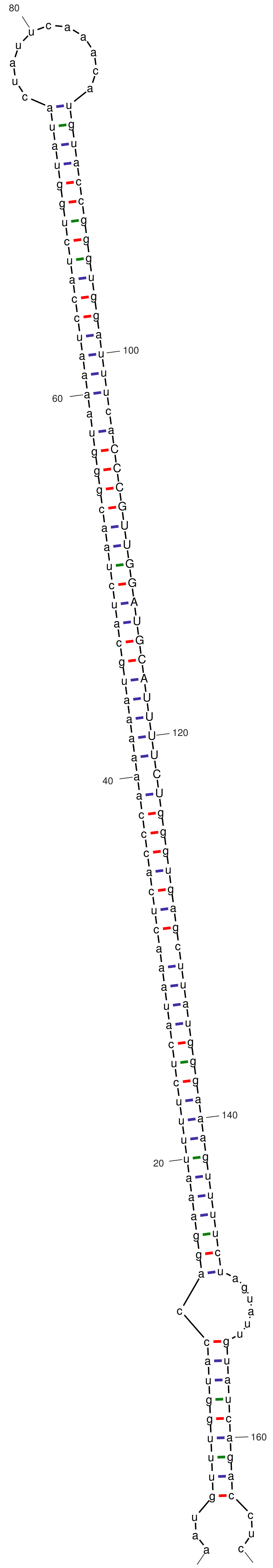

dG = -95.00 [Initially -95.00] miR_n07

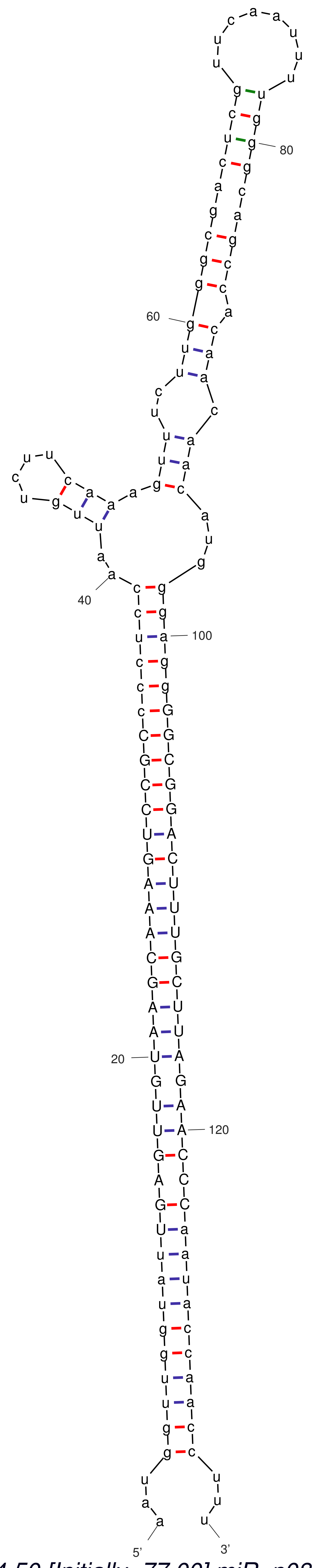

dG = -74.50 [Initially -77.00] miR_n08-5p

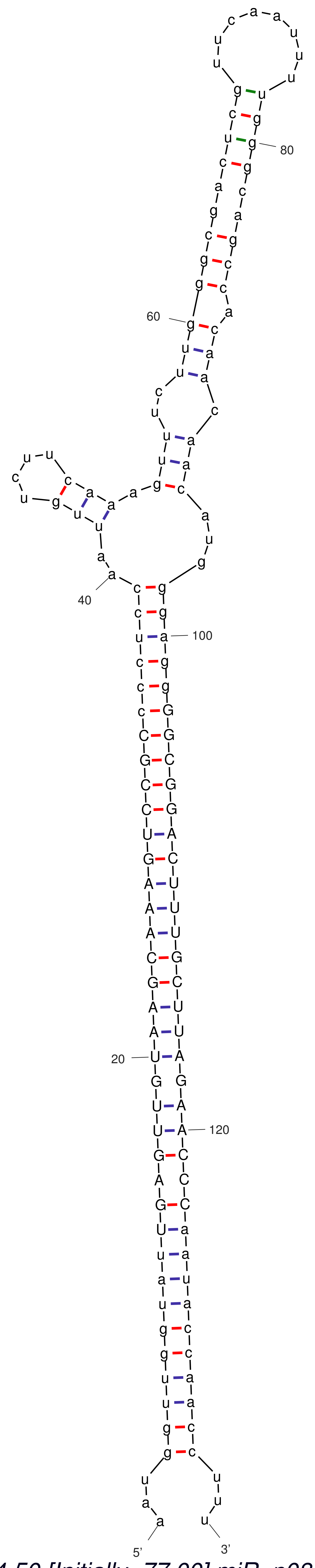

dG = -74.50 [Initially -77.00] miR_n08-3p

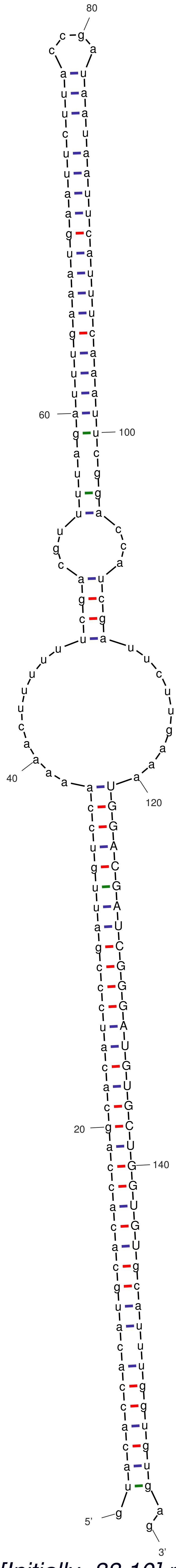

dG = -88.10 [Initially -88.10] miR_n09

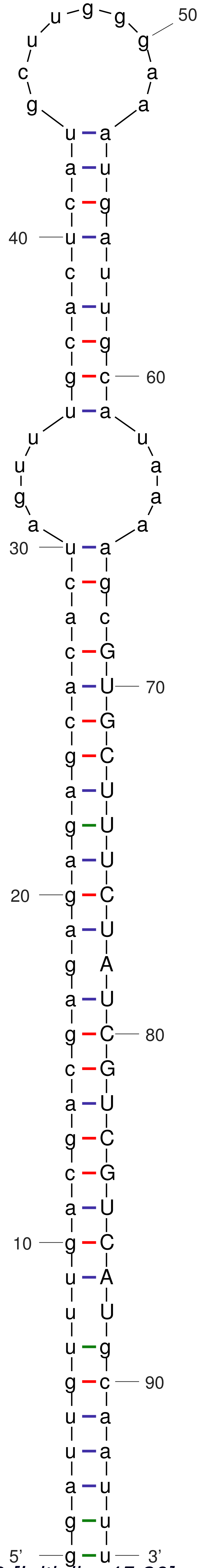

dG = -45.70 [Initially -45.20] miR_n10

Output of sir_graph (©)
mfold_util 4.7

Created Sat Dec 24 08:41:34 2016

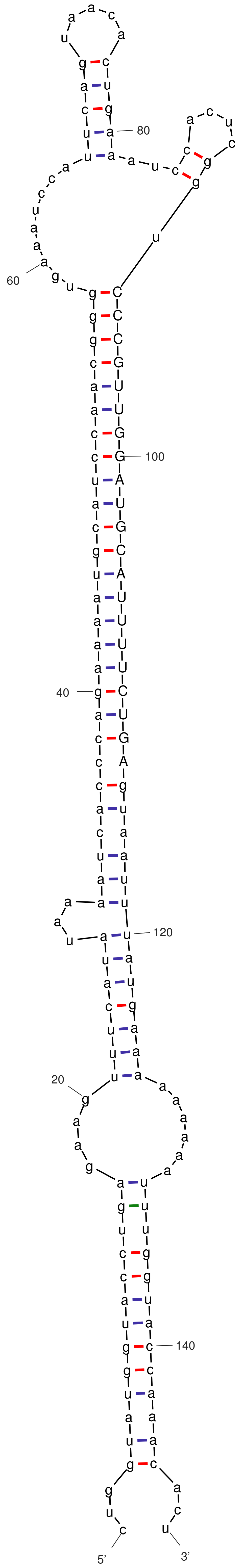

dG = -56.34 [Initially -60.10] miR_n11

Output of sir_graph (©)
mfold_util 4.7

Created Sat Dec 24 08:42:05 2016

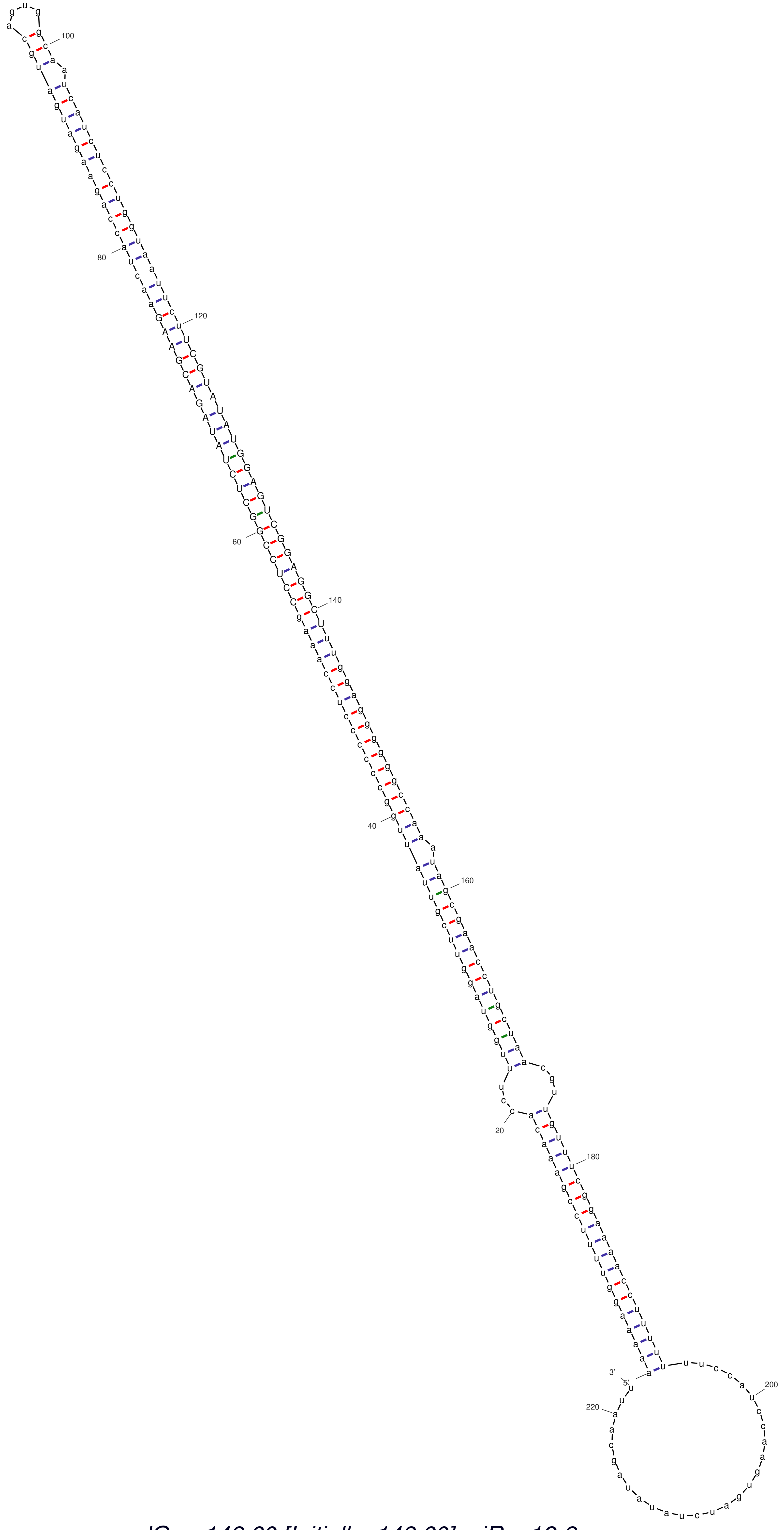

dG = -148.60 [Initially -148.60] miR_n12-3p

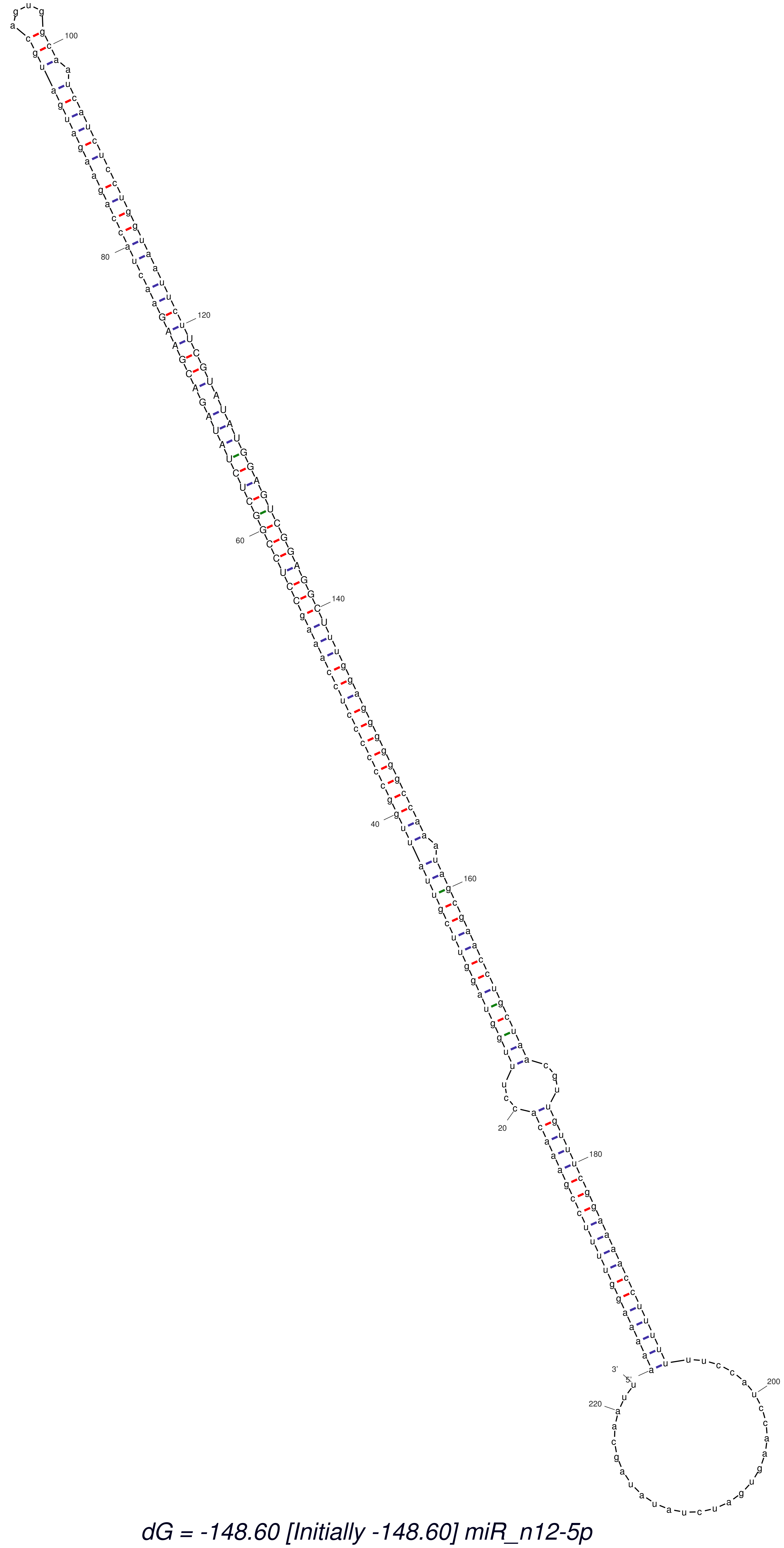

dG = -148.60 [Initially -148.60] miR_n12-5p

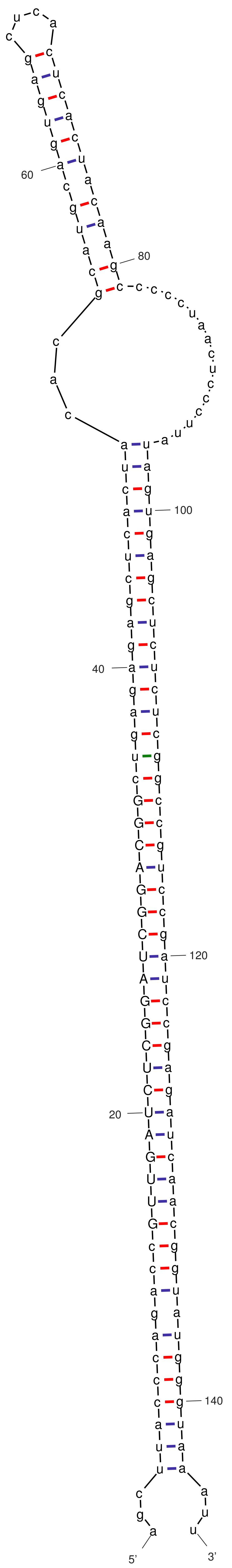

dG = -101.80 [Initially -101.80] miR_n13

Output of sir_graph (©)
mfold_util 4.7

Created Sat Dec 24 08:43:59 2016

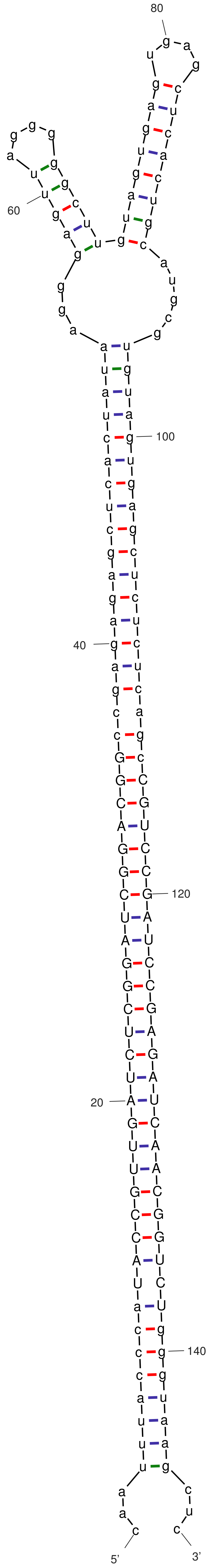

dG = -104.98 [Initially -107.80] miR_n14
