# Supplementary material for: Comparative Analysis of Fruit Ripening-Related miRNAs and Their Targets in Blueberry Using Small RNA and Degradome Sequencing
Source: Int J Mol Sci. 2017 Dec 19;18(12):2767. doi: 10.3390/ijms18122767 (PMC5751366; doi:10.3390/ijms18122767)
Supplement: Supplementary file 1 [file ijms-18-02767-s001.zip › Captions.docx]

Table S1. Summary information of known miRNAs identified from BF and WF libraries in blueberry.

Table S2. Identified targets for miRNAs by degradome sequencing in blueberry.

Table S3. Information of vco-miRNAs targets by degradome sequencing.

Table S4. The sequences of specific stem-loop RT and qRT-PCR primers for miRNAs.

Table S5. The sequences of target gene specific primers for qRT-PCR analysis.

Table S6 The sequences of gene specific primers for RLM-5’ RACE analysis.

Figure S1. Length distribution of small RNAs in blue fruit stage (BF) and white fruit stage (WF) libraries from blueberry fruit.

Figure S2. Proportion of nucleotide bias at each position within total miRNAs in a library of small RNAs from blueberry fruit.

Figure S3. Secondary structures of the novel pre-miRNAs in blueberry. Mature miRNA sequences are highlighted in yellow.

Figure S4. Phylogenetic analysis of known pre-miRNA sequences of blueberry (*Vaccinium corymbosum*) and grape (*Vitis vinifera*) obtained from sequencing data and miRbase 21.0, respectively. The pre-miRNAs of blueberry are marked with star. The backgrounds in different colors show the different miRNA families.

Figure S5. Target plot (t-plots) of blueberry miRNA targets and validation of target genes by RLM 5’-RACE. Representative t-plots for category 0 (A-D), category 1 (E), category 2 (F, G), category 3 (H) and category 4 (I) are shown. The red line represents the sliced target transcripts. miRNA: mRNA alignments along with the miRNA-directed cleavage positions are shown below. The vertical arrow indicates the exact cleavage site and the numbers show the frequency of clones sequenced.
