# Supplementary figures and images for: Comparative Analysis of Fruit Ripening-Related miRNAs and Their Targets in Blueberry Using Small RNA and Degradome Sequencing
Source: Int J Mol Sci. 2017 Dec 19;18(12):2767. doi: 10.3390/ijms18122767 (PMC5751366; doi:10.3390/ijms18122767)

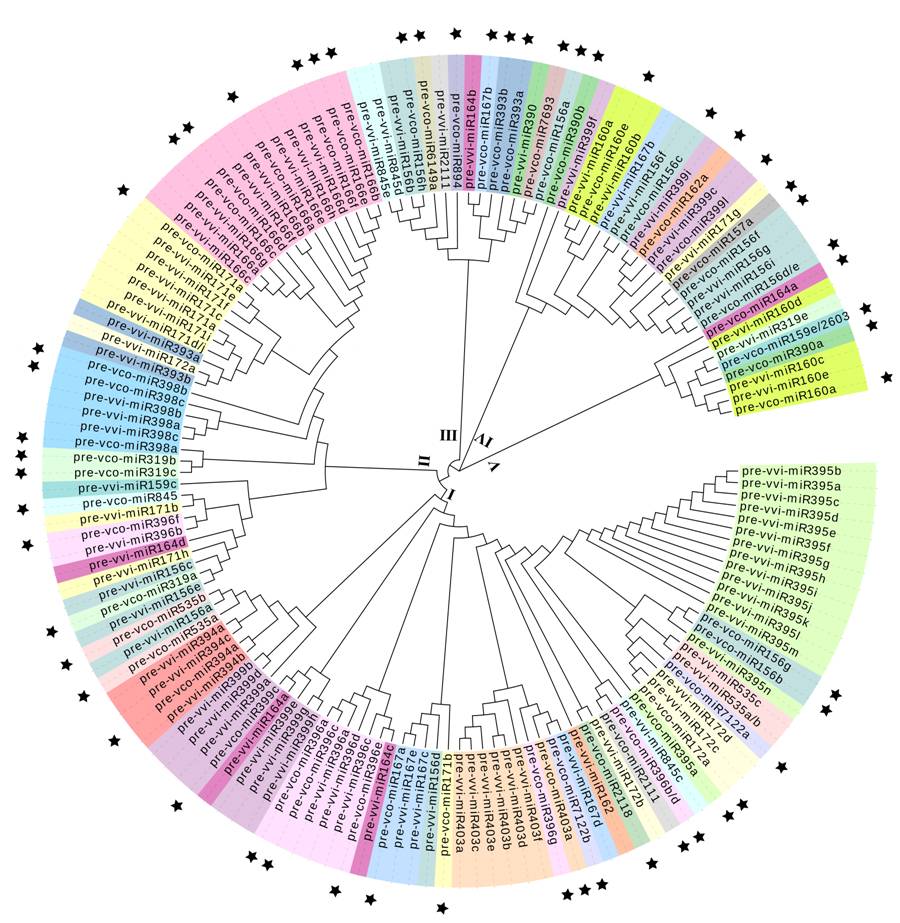

Supplement: Supplementary file 1 [file ijms-18-02767-s001.zip › Figure S4.jpg]

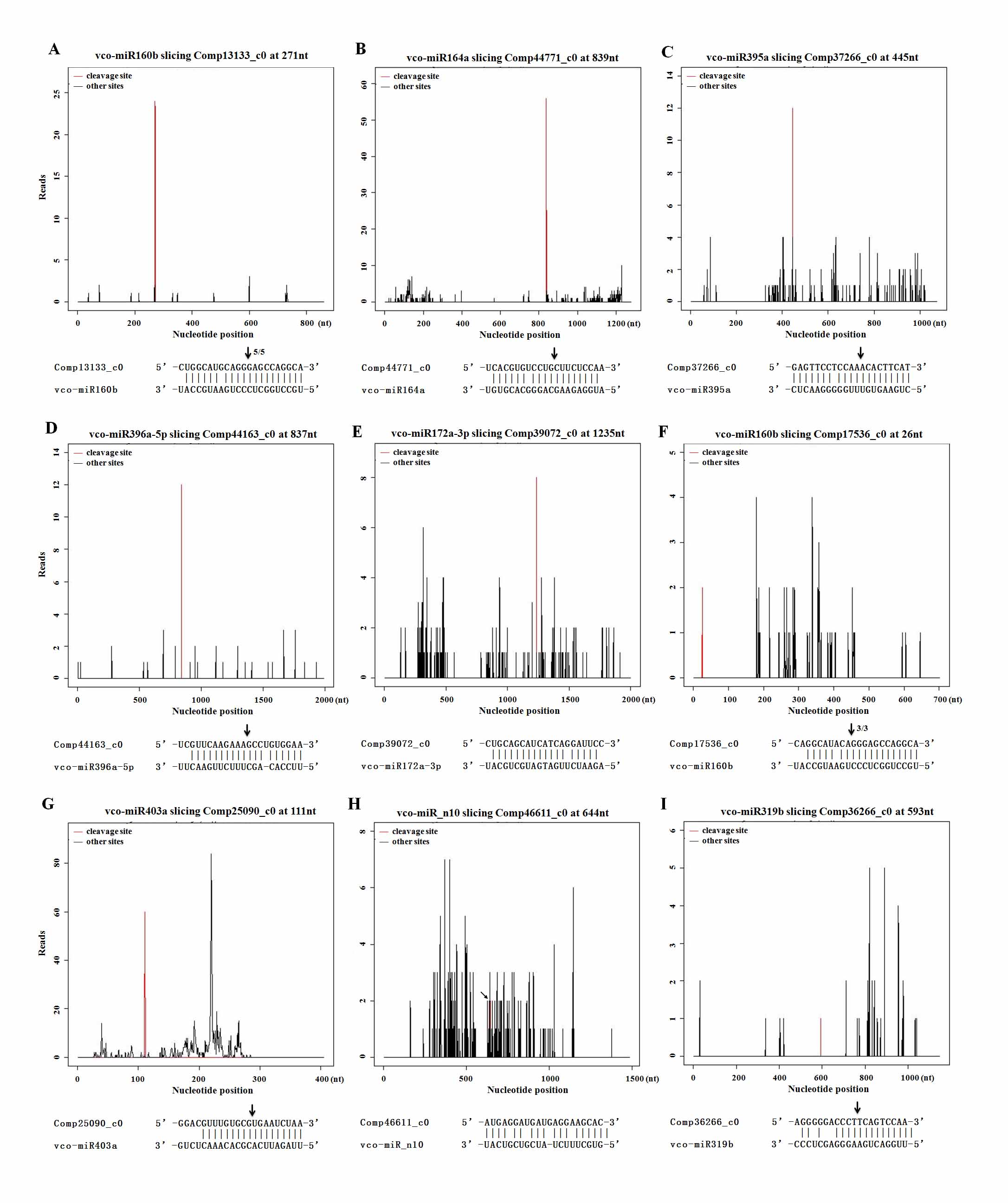

Supplement: Supplementary file 1 [file ijms-18-02767-s001.zip › Figure S5.jpg]

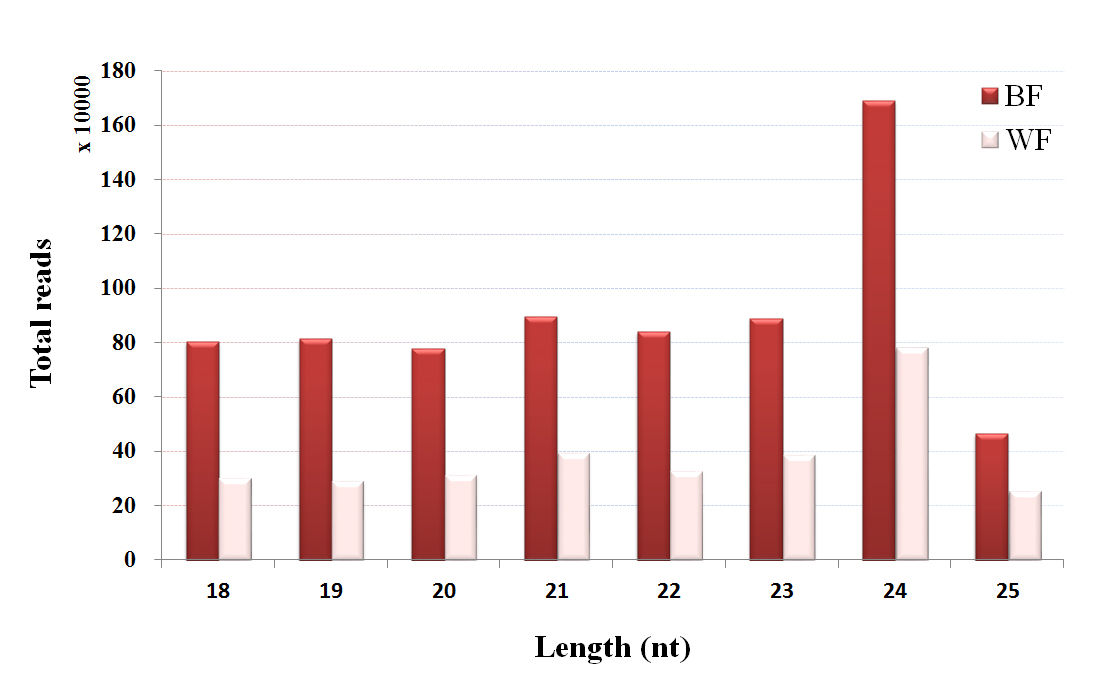

Supplement: Supplementary file 1 [file ijms-18-02767-s001.zip › Figure S1.jpg]

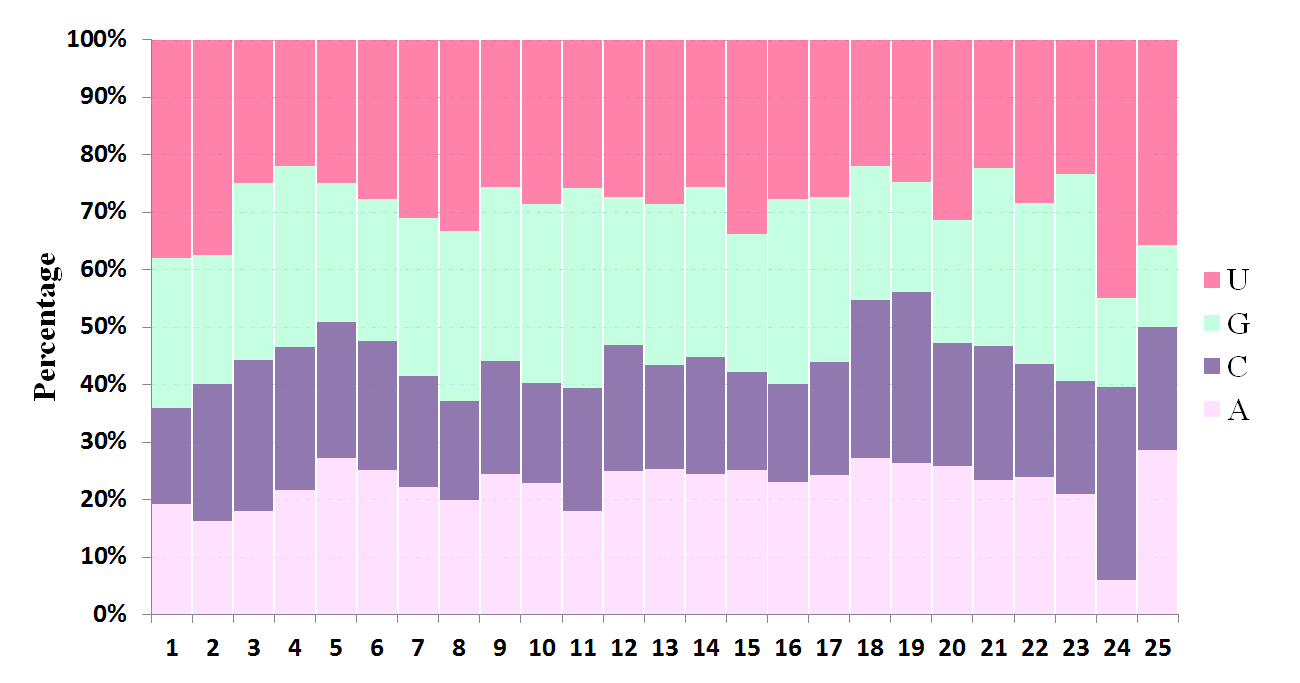

Supplement: Supplementary file 1 [file ijms-18-02767-s001.zip › Figure S2.jpg]
